# Supplementary material for: Hominoid SVA-lncRNA AK057321 targets human-specific SVA retrotransposons in SCN8A and CDK5RAP2 to initiate neuronal maturation
Source: Commun Biol. 2023 Mar 30;6:347. doi: 10.1038/s42003-023-04683-8 (PMC10063665; doi:10.1038/s42003-023-04683-8)
Supplement: Supplementary file 2 — Supplementary Information [file 42003_2023_4683_MOESM2_ESM.pdf]

SUPPLEMENTARY INFORMATION

Hominoid SVA-lncRNA *AK057321* targets human-specific SVA retrotransposons in *SCN8A* and *CDK5RAP2* to initiate neuronal maturation

Authors: Monica J.S. Nadler[1,2], Weipang Chang[1,2], Ekim Ozkaynak[1,2], Yuda Huo[1,2,5], Yi Nong[1,2,5], Morgane Boillot[1,2], Mark Johnson[1,2], Antonio Moreno[1,2], and Matthew P. Anderson‡ [1,2,3,4,5]

<u>**Supplementary Figures 1-21**</u>

Supplementary Fig. 1: Example long non-coding RNAs which contain SVA transposon including *AK057321*.

Supplementary Fig. 2: Expression of full-length *AK057321* human gene in a transgenic mouse compared to wild-type mice.

Supplementary Fig. 3: SVA-lncRNA *AK057321* levels increase as iPSC cells are differentiated into excitatory (glutamatergic), but not cholinergic or dopaminergic neurons.

Supplementary Fig. 4: Example microcephaly genes with intronic SVAs.

Supplementary Fig. 5: Example neuronal dendrite and axon growth genes with intronic SVAs.

Supplementary Fig. 6. Example synapse organizing genes within intronic SVAs.

Supplementary Fig. 7: Example ion channel genes that contain SVA transposons.

Supplementary Fig. 8: Complete gel image for Fig. 1h, deletion of the SVA_B within SVA-lncRNA *AK057321*.

Supplementary Fig. 9: Outward Potassium currents are larger in *AK057321* OE and *ZNF91* sgRNA treatment groups measured during depolarizing voltage steps.

Supplementary Fig. 10: Additional quantification of neuronal morphology of Ntera-2 cells with or without *AK057321* OE**.**

Supplementary Fig. 11: Complete gel image for deletion of SVA_F in *CDK5RAP2*.

Supplementary Fig. 12: Effects of SVA-lncRNA *AK057321* and other interventions on *SCN8A* expression and tetrodotoxin-inhibited sodium spikes.

Supplementary Fig. 13: Complete gel image for deletion of the intronic SVA_D within *SCN8A*.

Supplementary Fig. 14: Deleting the SVA in *CHAF1B* increases stemness gene transcripts and overall cell number recovery.
Supplementary Fig. 15: Complete gel image for deletion of the intronic SVA_B within *CHAF1B*.

Supplementary Fig. 16: SVA-lncRNA *AK057321* expression is enriched in cortex and cerebellum relative to subcortex and brainstem expressed as mouse transgene.

Supplementary Fig. 17: *AK057321* fails to regulated human chromosome 21 genes lacking SVAs.

Supplementary Fig. 18: Multiple human genes with intronic SVAs are up-regulated in human relative to mouse cerebellum and are upregulated by SVA-lncRNA *AK057321* in mouse 3T3 fibroblast stably expressing these human genes (bacterial artificial chromosome, BAC vector).

Supplementary Fig. 19: Transcriptional repression by intronic SVA transposons and ZNF91 transcription factor is reversed fully by VNTR repeat deletion (in *CHAF1B* SVA) and partially by SVA-lncRNA *AK057321*.

Supplementary Fig. 20: 5'-apatamer-tagged SVA-lncRNA *AK057321* binds to streptavidin beads.

Supplementary Fig. 21: Complete gel image for 5'-apatamer-tagged SVA-lncRNA *AK057321* binds to streptavidin beads.

Supplementary Tables 1-20

Supplementary Table 1. SVA-lncRNA *AK057321* shRNA target sequences and cloning primers.

Supplementary Table 2. Construction primers used to modify lentiCRISPRv2 hygro (Addgene #98291) to express in tandem sgRNAs with Cas9 and either mcherry or blue fluorescent protein.

Supplementary Table 3. sgRNA sequences used to delete SVAs.

Supplementary Table 4. Genomic PCR primers used to screen for SVA deletions.

Supplementary Table 5. Dual aptamer tag sequence.

Supplementary Table 6. Primers used to generate Tet-inducible *CDK5RAP2* cDNA.

Supplementary Table 7. Primers used to clone *CHAF1B* SVA into pGL3 luciferase vector.

Supplementary Table 8. Primers used to generate *CHAF1B* SVA minusVNTR sequence into pGL3 luciferase vector.

Supplementary Table 9. Primers used to clone *HTT* SVA into pGL3 luciferase vector.

Supplementary Table 10. Primers used to clone *JAM2*, *AGPAT3*, *POFUT2* and *CDK5RAP2* SVAs.

Supplementary Table 11. Table of BAC clone numbers used in this study.

Supplementary Table 12. Table of primers used in BAC recombineering.

Supplementary Table 13. Table of BAC screening primers.

Supplementary Table 14. Primers used in BAC recombineering to delete the SVA within *CHAF1B* on BAC clone RP11-108J14.

Supplementary Table 15. Primers used to clone zeocin resistance gene.

Supplementary Table 16. Table of IDT primers and probes used in gene expression studies.

Supplementary Table 17. Table of Eurofin primers used in gene expression studies.

Supplementary Table 18. Primers used for relative quantitative PCR (rqPCR) for *AK057321* expression analysis in Supplementary Fig. 12.

Supplementary Table 19. Thermocycler conditions for reverse transcriptase and polymerase chain reactions.

Supplementary Table 20. Genomic IDT primers and probes used in SVA-lncRNA *AK057321* binding studies.

Supplementary Data 1-6 (excel spreadsheets)

Supplementary Data 1. Duplication of SVA-lncRNA *AK057321* in rare cases of autism spectrum disorder.

Supplementary Data 2. Source data for main manuscript Figure graphs.

Supplementary Data 3. Source data for supplementary manuscript Figure graphs.

Supplementary Data 4. Human genes containing intronic SVAs.

Supplementary Data 5. Gene ontology disease enrichment of human genes with intragenic SVAs.

Supplementary Data 6. Gene ontology term enrichment of human genes with intragenic SVAs.

Supplementary Figures 1-21

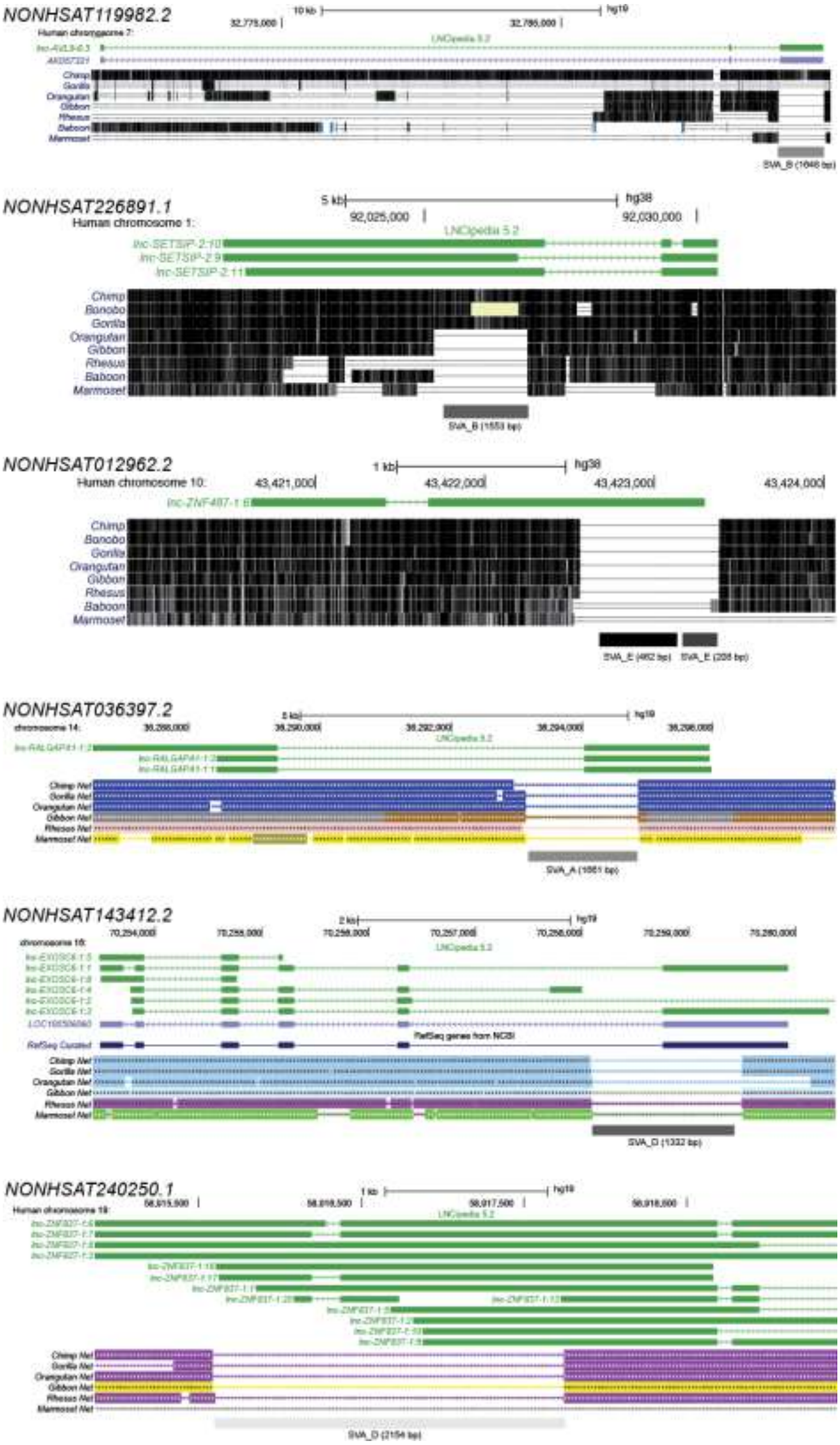

Supplementary Fig. 1: Example long non-coding RNAs which contain SVA transposon including SVA-lncRNA *AK057321* (NONSAT119982.2) (UCSC genome browser, GRCh38/gh38).

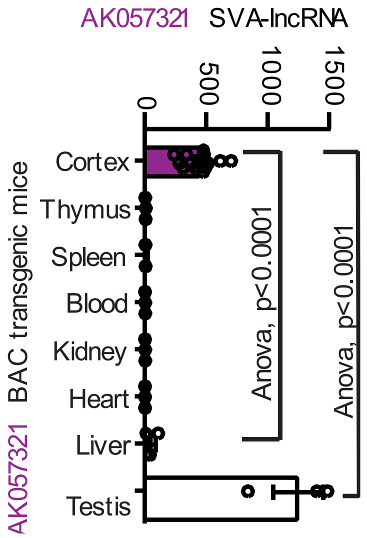

Supplementary Fig. 2: Expression of full-length SVA-lncRNA *AK057321* human gene in a transgenic mouse compared to wild-type mice (*GAPDH*, reference; n=14 biologicals for cortex and 1 for other tissue, measured in triplicate). One-way ANOVA ($F_{6,24}$=26.14, p<0.0001; excluding testis) and ($F_{7,26}$ =34.72, p<0.0001 with all tissues). Data represent the mean ± SEM.

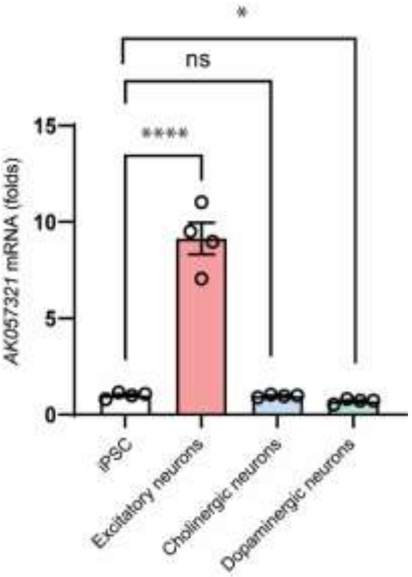

Supplementary Fig. 3: SVA-lncRNA *AK057321* mRNA increases as iPSC cells are differentiated into excitatory (glutamatergic), but not cholinergic or dopaminergic neurons. RNA from each cell type was obtained from Elixirgen Scientific and gene expression was determined by RT-qPCR with *GAPDH* as the internal reference gene. Unpaired Student's t test. Data represent the mean ± SEM.**** p<0.0001, *** p<0.001, ** p<0.01, * p<0.05 and ns p>0.05 versus control.

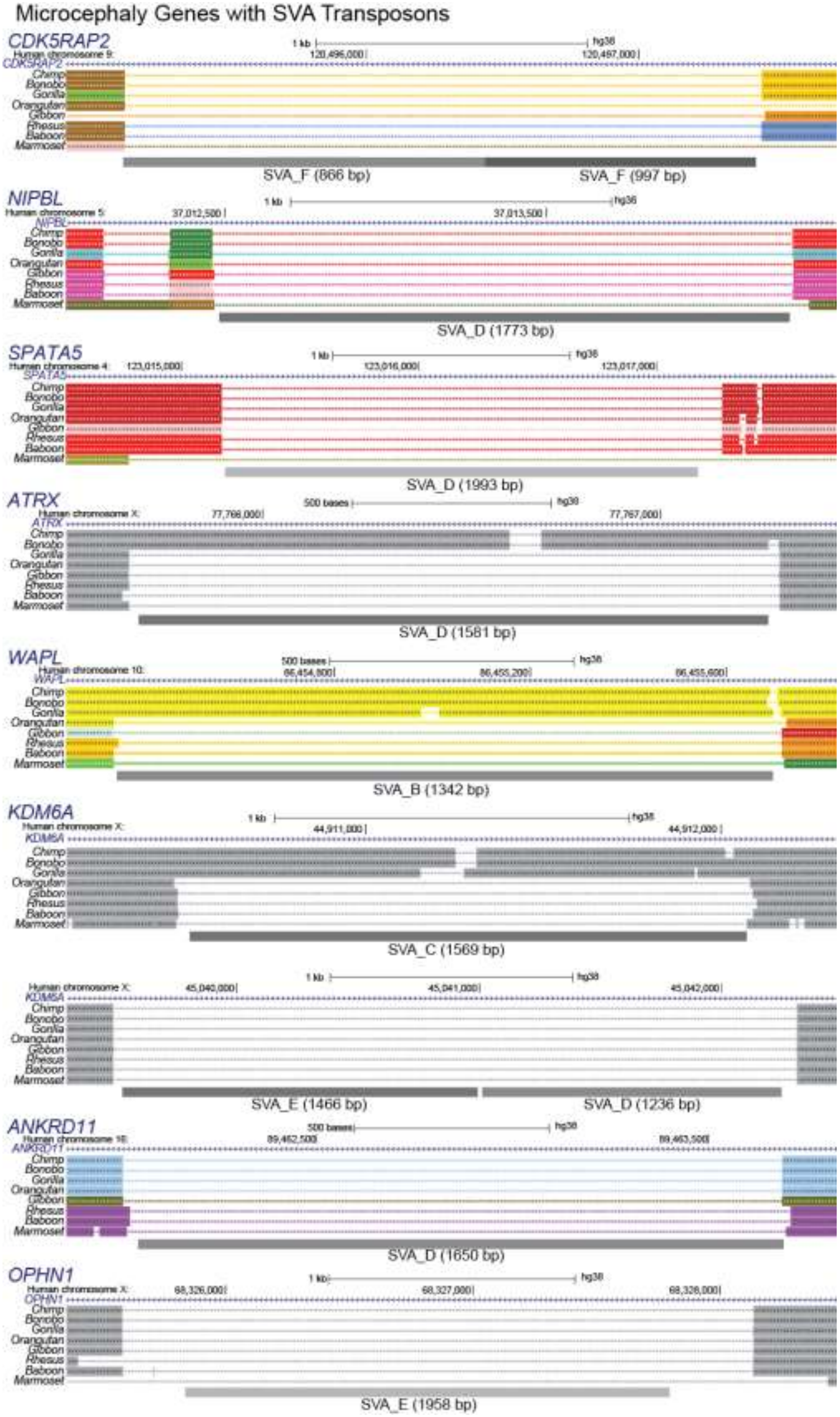

Supplementary Fig. 4: Example microcephaly genes with intronic SVAs (UCSC genome browser, GRCh38/gh38).

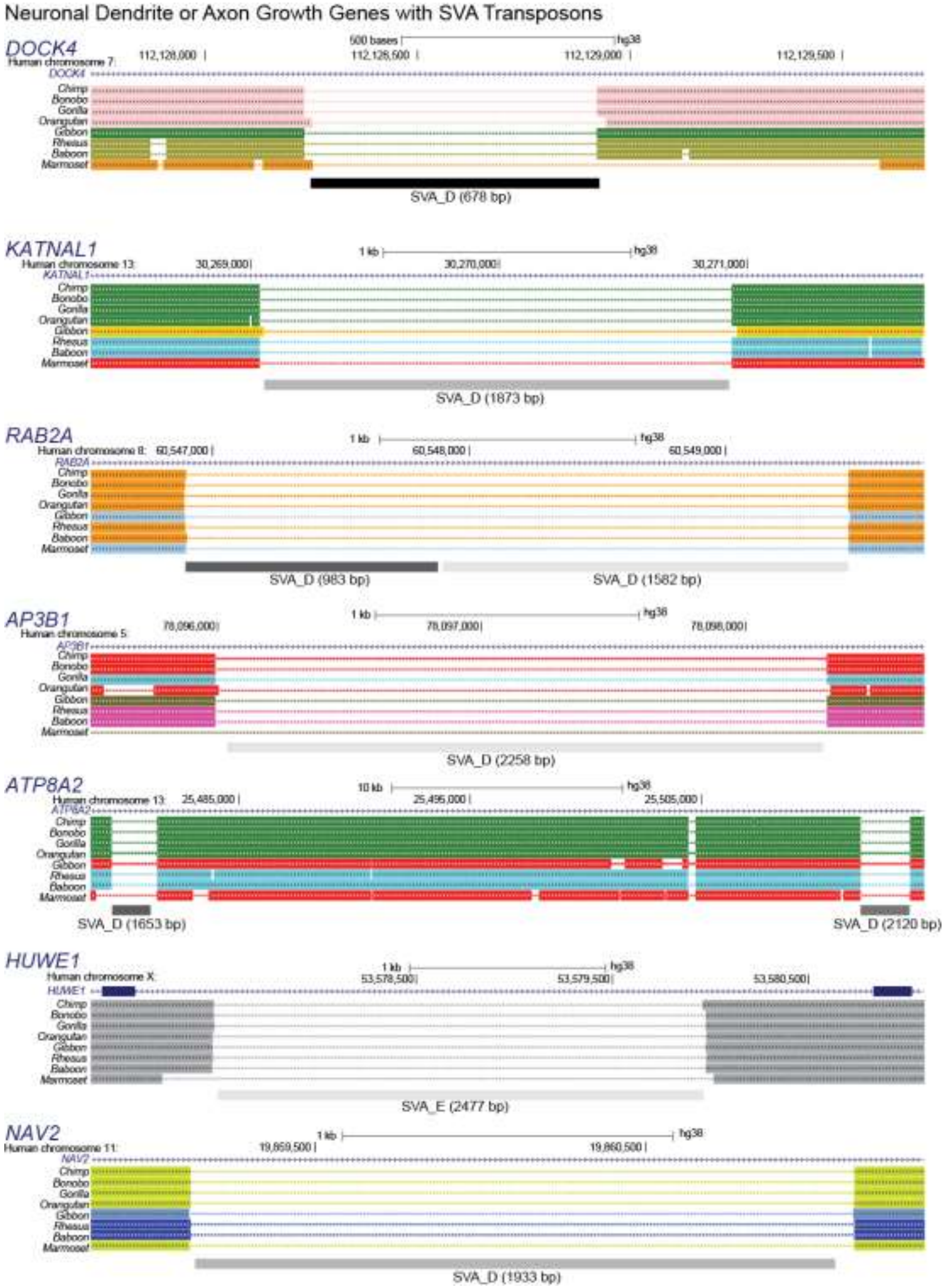

Supplementary Fig. 5: Example neuronal dendrite and axon growth genes with intronic SVAs (UCSC genome browser, GRCh38/gh38).

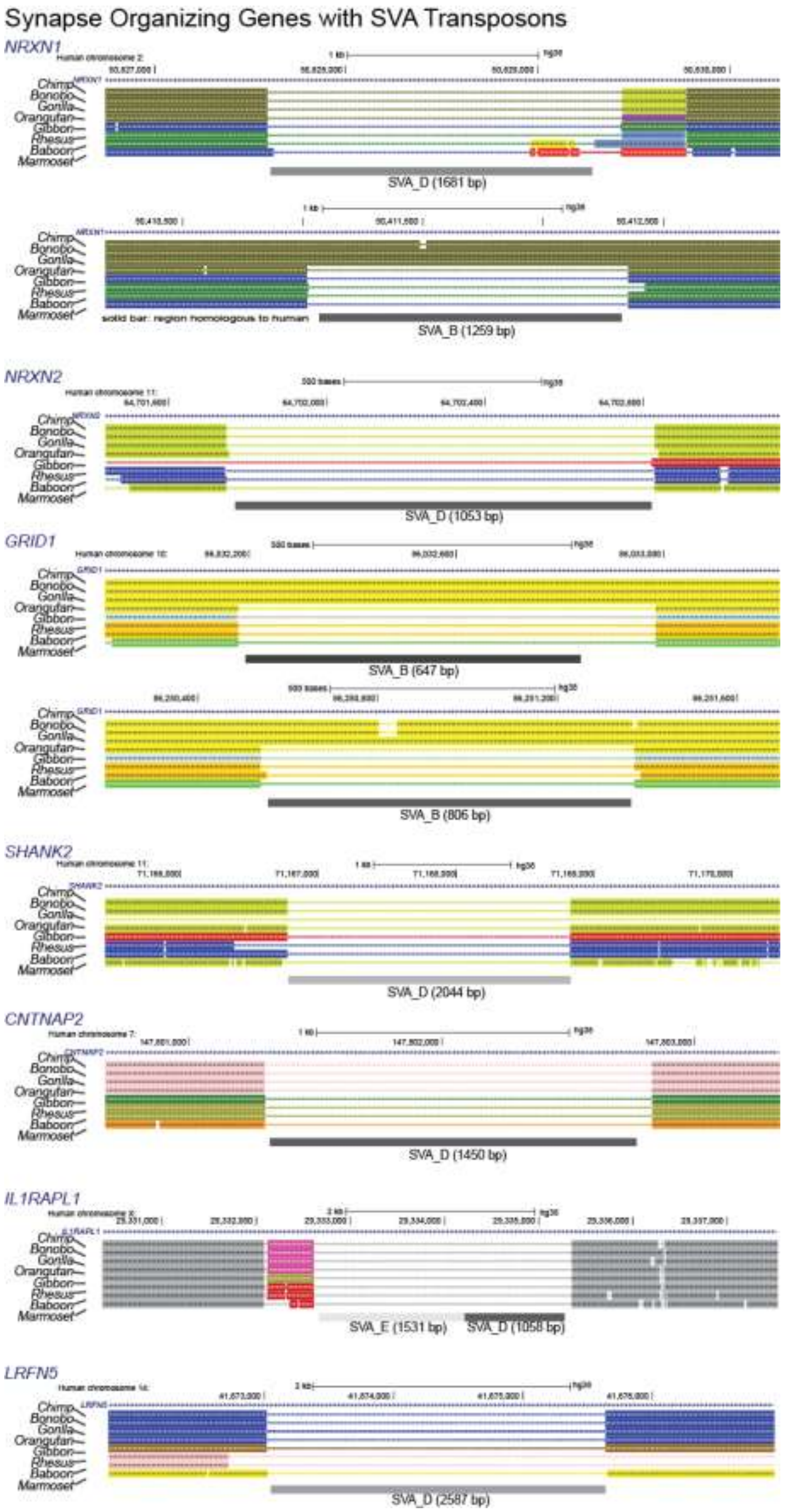

Supplementary Fig. 6. Example synapse organizing genes within intronic SVAs (UCSC genome browser, GRCh38/gh38).

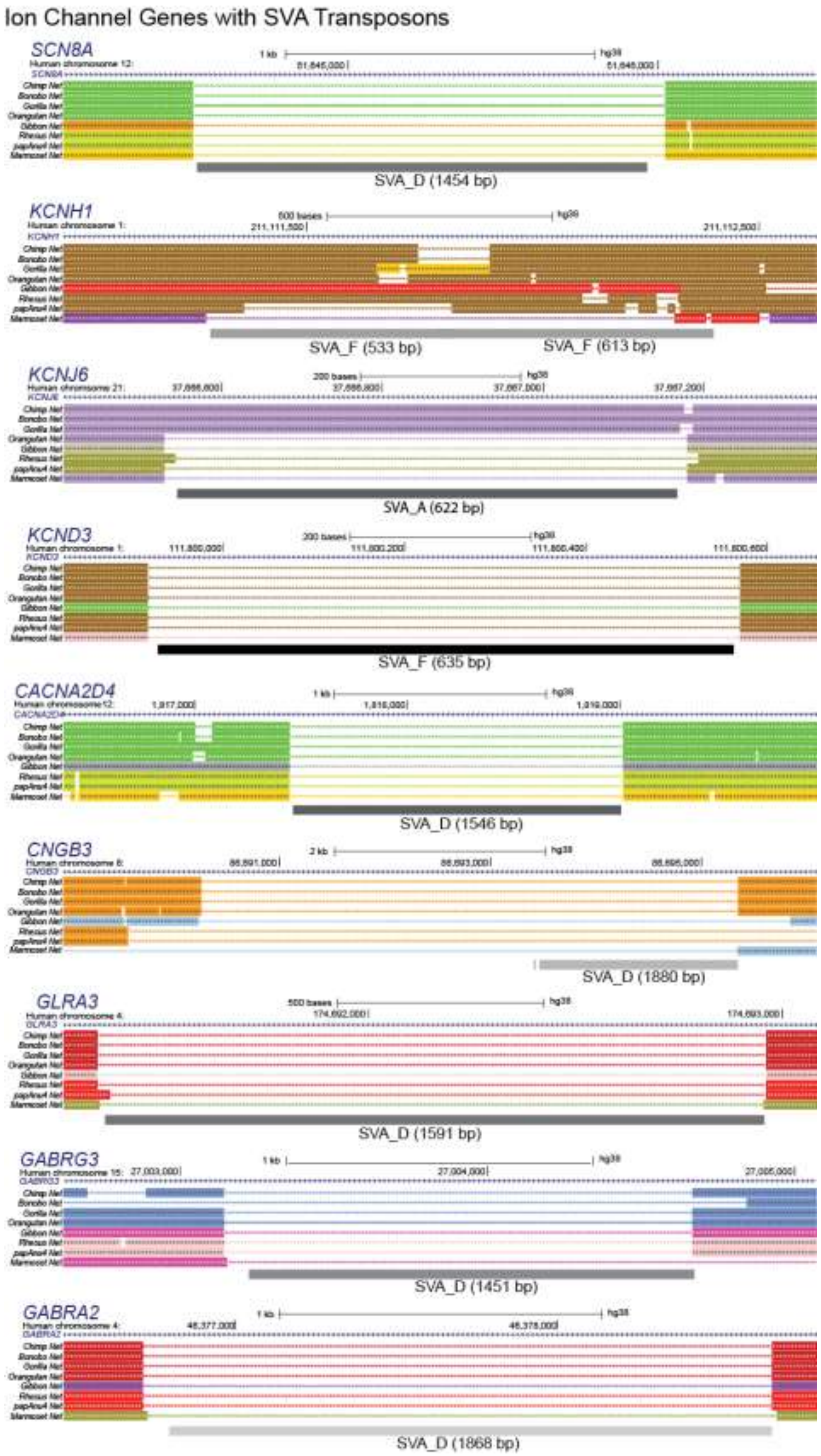

Supplementary Fig. 7: Example ion channel genes that contain SVA transposons (UCSC genome browser, GRCh38/gh38).

a

Lane	Expected size	PCR primers	Cas9/sgRNA transfection
1	DNA ladder	NEB 1kb Plus DNA ladder	NA
2	2870 bp	*AK057321* del SVA F1 & R1	Cas9/sgRNA control
3	309 bp	*AK057321* del SVA F1 & R1	Set 1 guides
4	302 bp	*AK057321* del SVA F1 & R1	Set 2 guides
5	365 bp	*AK057321* del SVA F1 & R1	Set 3 guides
6	2870 bp	*AK057321* del SVA F1 & R1	Ntera-2 no transfection
7	none	*AK057321* del SVA F1 & R1	None – water control
8	2870 bp	*AK057321* del SVA F1 & R1	1:10 Cas9/sgRNA control gDNA
9	309 bp	*AK057321* del SVA F1 & R1	1:10 dilution Set 1 guide gDNA
10	302 bp	*AK057321* del SVA F1 & R1	1:10 dilution Set 2 guide gDNA
11	364 bp	*AK057321* del SVA F1 & R1	1: 10 dilution Set 3 guide gDNA
12	2870 bp	*AK057321* del SVA F1 & R1	1:10 dilution Ntera-2 gDNA
13	none	*AK057321* del SVA F1 & R1	None – water control

b

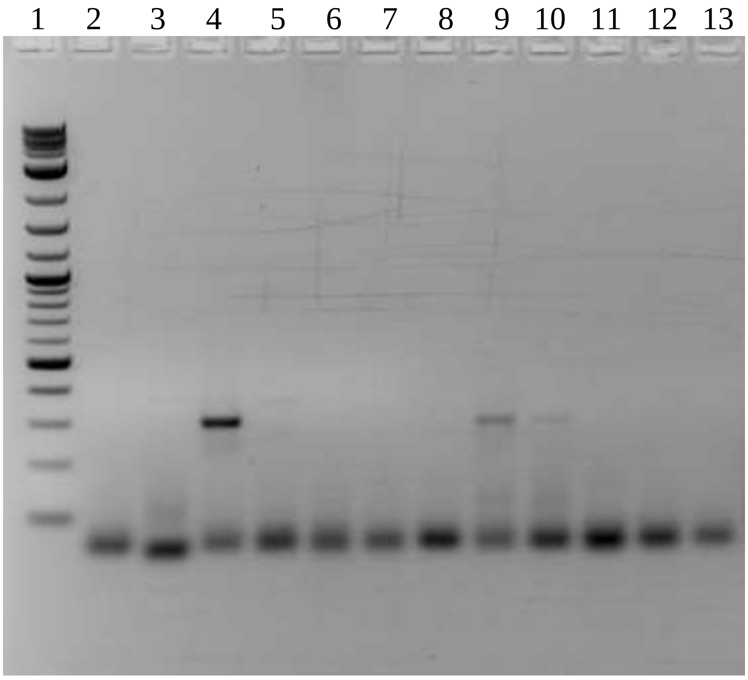

Supplementary Fig. 8: Complete gel image for **Fig. 1h**, deletion of the SVA_B within SVA-lncRNA *AK057321*. **a**, Table for the genomic samples and primers used to detect deletion of the SVA within *AK057321* for the uncropped gel image in **b** that was used to generate **Fig. 1h**. All sgRNA combinations and sequences are in Supplementary Table 3 and the genomic PCR primer sequences are listed in Supplementary Table 4. Set 2 guides showed evidence of deletion of the SVA based on the expected band size of 302 bp, lanes 4 and 10. Note that set 1 guides also showed a band of the expected size 309 bp in lane 9. The undeleted PCR band (i.e. band spanning the SVA) was not visualized in lanes 2, 6, 8 and 12 because of the short PCR extension time.

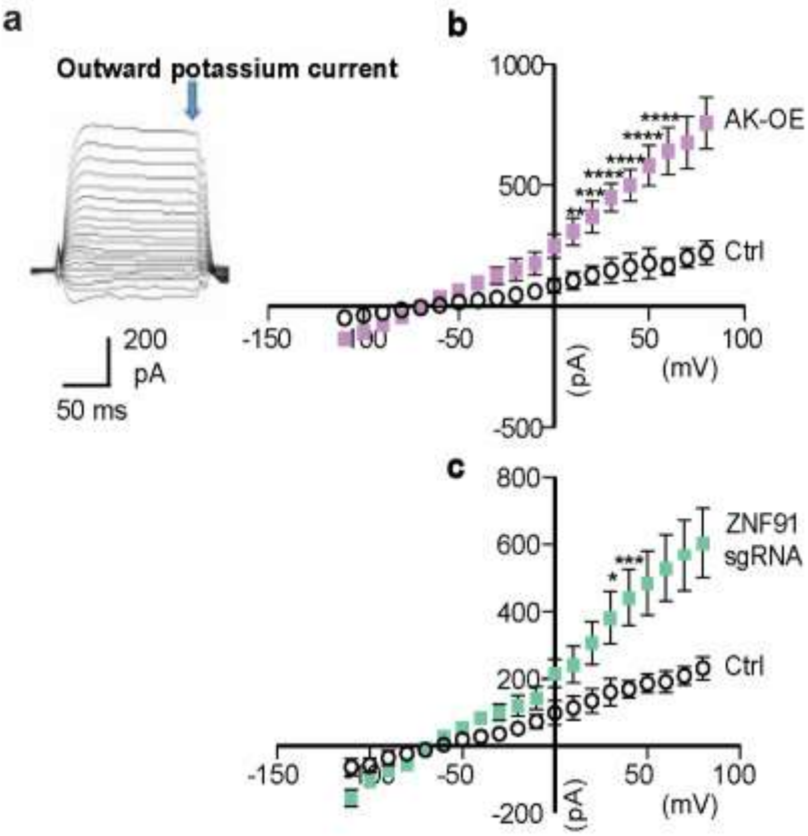

Supplementary Fig. 9: Outward Potassium currents are larger in SVA-lncRNA *AK057321* overexpressing (AK-OE) and *ZNF91* sgRNA treatment groups measured during depolarizing voltage steps. **a**, Current responses to voltage steps in different treatment groups. **b** and **c**, Total Ik currents were significantly larger in *AK057321* OE (n=6) and *ZNF91* sgRNA (n=6) groups than that in the control group**.** Two-way ANOVA with Bonferroni post hoc tests. Data represent the mean ± SEM. **** $p<0.0001$, ** $p<0.01$.

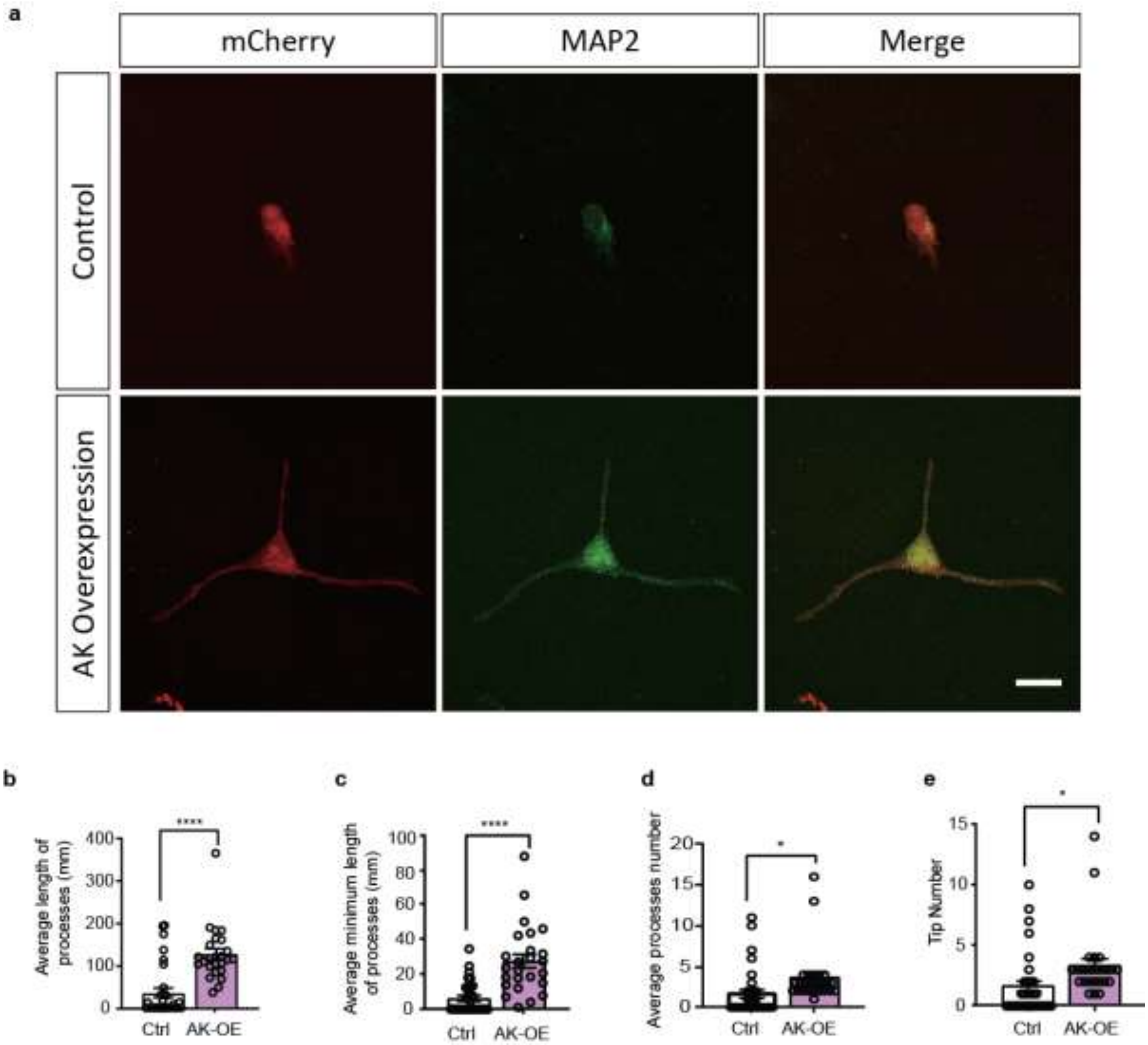

Supplementary Fig. 10: Additional quantification of neuronal morphology of NTera-2 cells with or without SVA-lncRNA *AK057321* overexpression (AK-OE). **a**, NTera-2 cells transfected with control or AK overexpression plasmids (containing mCherry reporter, red fluorescence), and 13 days after plating, the cells were fixed, permeabilization and stained with MAP2 antibody (green immunofluorescence). Scale bar is 20 microns. **b**, Quantification of average mean length of processes from NTera-2 cells with or without *AK057321* OE. **c**, Quantification of average minimum length of processes. **d**, Quantification of average process number. **e**, Quantification of tip number. All graphs are presented as Mean ± SEM. Control, N = 31, AK-OE, N = 25. Unpaired two-tailed Student's t test. Data represent the mean ± SEM.**** p<0.0001, *** p<0.001, ** p<0.01, * p<0.05 versus control.

a

Lane	Expected size	PCR primers	Cas9/sgRNA transfection
1	DNA ladder	NEB 1kb Plus DNA ladder	NA
2	1255 bp	*CDK5RAP2* del SVA F1 and R1	Set 1 guides
3	1230 bp	*CDK5RAP2* del SVA F1 and R1	Set 2 guides
4	1188 bp	*CDK5RAP2* del SVA F1 and R1	Set 3 guides
5	1757 bp	*CDK5RAP2* del SVA F1 and R1	Set 4 guides
6	1787 bp	*CDK5RAP2* del SVA F1 and R1	Set 5 guides
7	none	*CDK5RAP2* del SVA F1 and R1	None – water control
8	4372 bp	*CDK5RAP2* del SVA F2 and R1	Ntera-2 no transfection
9	4372 bp	*CDK5RAP2* del SVA F2 and R1	Cas9/sgRNA control
10	993 bp	*CDK5RAP2* del SVA F2 and R1	Set 1 guides
11	968 bp	*CDK5RAP2* del SVA F2 and R1	Set 2 guides
12	926 bp	*CDK5RAP2* del SVA F2 and R1	Set 3 guides
13	1495 bp	*CDK5RAP2* del SVA F2 and R1	Set 4 guides
14	1525 bp	*CDK5RAP2* del SVA F2 and R1	Set 5 guides
15	none	*CDK5RAP2* del SVA F2 and R1	None – water control
16	4677 bp	*CDK5RAP2* del SVA F3 and R1	Ntera-2 no transfection
17	4677 bp	*CDK5RAP2* del SVA F3 and R1	Cas9/sgRNA control
18	1298 bp	*CDK5RAP2* del SVA F3 and R1	Set 1 guides
19	1273 bp	*CDK5RAP2* del SVA F3 and R1	Set 2 guides
20	DNA ladder	NEB 1kb Plus DNA ladder	NA
21	DNA ladder	NEB 1kb Plus DNA ladder	NA
22	1231 bp	*CDK5RAP2* del SVA F3 and R1	Set 3 guides
23	1800 bp	*CDK5RAP2* del SVA F3 and R1	Set 4 guides
24	1830 bp	*CDK5RAP2* del SVA F3 and R1	Set 5 guides
25	none	*CDK5RAP2* del SVA F3 and R1	None – water control
26	4461 bp	*CDK5RAP2* del SVA F4 and R2	Ntera-2 no transfection
27	4461 bp	*CDK5RAP2* del SVA F4 and R2	Cas9/sgRNA control
28	1152 bp	*CDK5RAP2* del SVA F4 and R2	Set 1 guides
29	1057 bp	*CDK5RAP2* del SVA F4 and R2	Set 2 guides
30	1015 bp	*CDK5RAP2* del SVA F4 and R2	Set 3 guides
31	1584 bp	*CDK5RAP2* del SVA F4 and R2	Set 4 guides
32	1614 bp	*CDK5RAP2* del SVA F4 and R2	Set 5 guides
33	none	*CDK5RAP2* del SVA F4 and R2	None – water control
34	4531 bp	*CDK5RAP2* del SVA F5 and R2	Ntera-2 no transfection
35	4531 bp	*CDK5RAP2* del SVA F5 and R2	Cas9/sgRNA control
36	1126 bp	*CDK5RAP2* del SVA F5 and R2	Set 1 guides
37	1127 bp	*CDK5RAP2* del SVA F5 and R2	Set 2 guides
38	1085 bp	*CDK5RAP2* del SVA F5 and R2	Set 3 guides
39	1654 bp	*CDK5RAP2* del SVA F5 and R2	Set 4 guides
40	DNA ladder	NEB 1kb Plus DNA ladder	NA

b

1 2 3 4 5 6 7 8 9 10 11 12 13 14 15 16 17 18 19 20

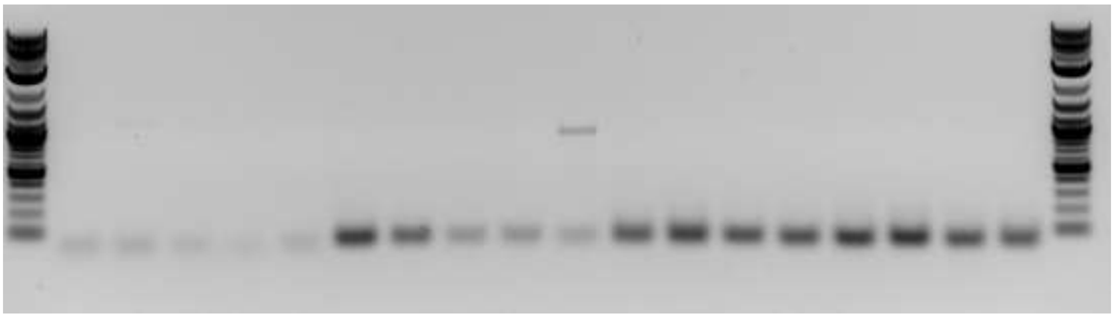

b (darker exposure)

1 2 3 4 5 6 7 8 9 10 11 12 13 14 15 16 17 18 19 20

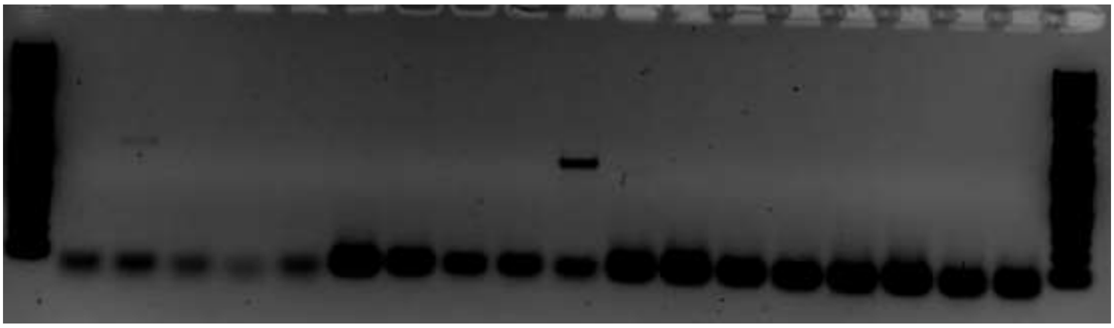

c

21 22 23 24 25 26 27 28 29 30 31 32 33 34 35 36 37 38 39 40

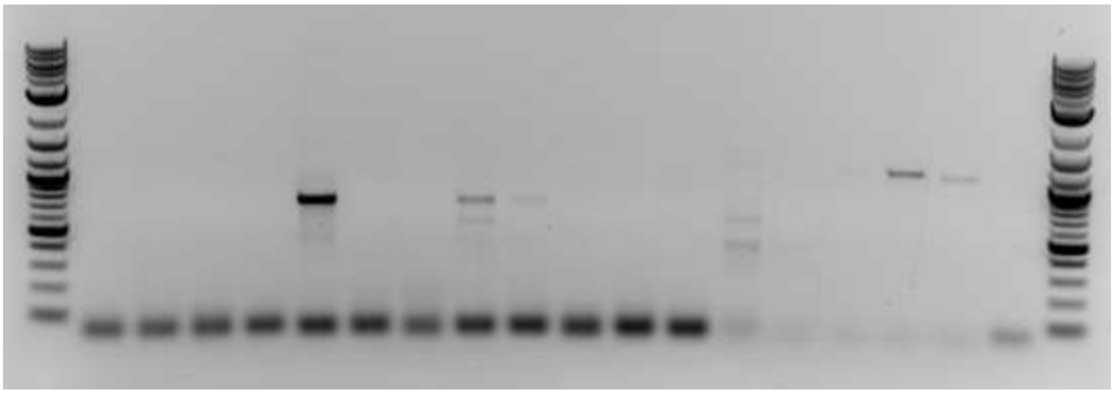

Supplementary Fig. 11: Complete gel image for deletion of the SVA_F within *CDK5RAP2*. **a**, Table to explain each lane shown in the uncropped and unedited DNA agarose gel images shown in **b** and **c**. Note that panel **b** is the parent image that was used to generate **Fig. 3b**, and panel **c** is additional data. All sgRNA combinations and sequences are in Supplementary Table 3 and all genomic PCR primer sequences are listed in Supplementary Table 4. Set 2 guides showed evidence of deletion of the SVA based on the expected band sizes obtained with different primer sets (i.e., lane 11, lane 37, and a weak band in lane 3). Note that set 3 guides also showed a band of the proper size in lane 38. Lanes 26, 27 and 30 had bands of incorrect sizes, and this screening primer combination of *CDK5RAP2* del SVA F4 and R2 was not used further. The undeleted PCR band (i.e., with the SVA is not visualized in lanes 8, 9, 16, 17, 26, 27, 34 and 35 because of the high GC content within the tandem SVA_F and because the PCR extension time was too short for bands over 2 Kb.

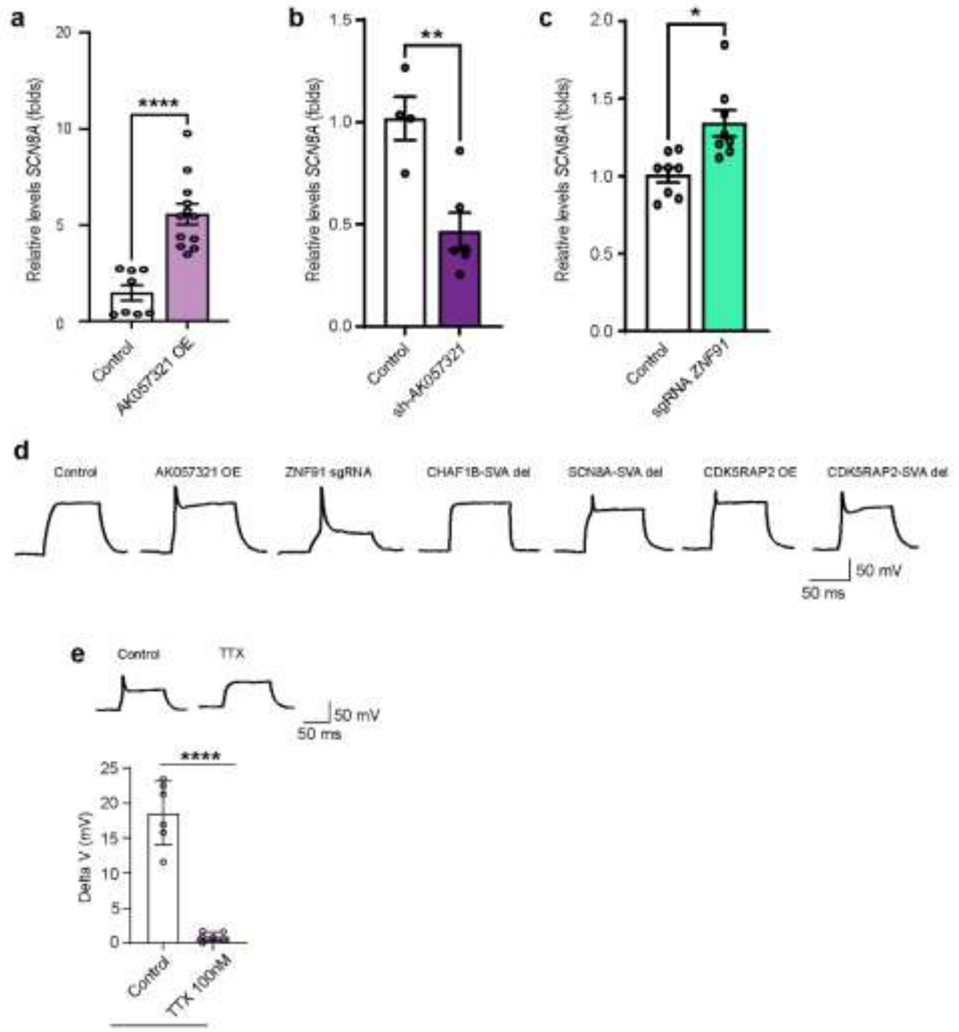

Supplementary Fig. 12: Effects of SVA-lncRNA *AK057321* and other interventions on *SCN8A* expression and tetrodotoxin-inhibited sodium spikes. **a**, RT-qPCR quantification of *SCN8A* mRNA in NTera-2 cells transfected to increased SVA-lncRNA *AK057321* expression (OE) or control plasmid with *GAPDH* as the internal reference gene. **b**, RT-qPCR quantification of *SCN8A* mRNA in NTera-2 cells transfected with either sh-*AK057321* or sh-scramble with *GAPDH* as the internal reference gene. **c**, RT-qPCR quantification of *SCN8A* mRNA in NTera-2 cells transfected with sgRNA/Cas9 *ZNF91* vs. control with *GAPDH* as the internal reference gene. Unpaired Student's two-tailed t test for individual gene comparisons to control in **a**-**c**. **d**, Example sodium spikes evoked in the *AK057321* OE, *ZNF91* sgRNA, *SCN8A*-SVA del, *CDK5RAP2* OE and *CDK5RAP2*-SVA del groups. 300 pA or larger current steps failed to induce sodium spikes in control and *CHAF1B*-SVA del groups. **e**, Evoked sodium spikes were blocked by 100 nM tetrodotoxin (TTX, n=7 in each group), .**** $p<0.0001$, *** $p<0.001$, ** $p<0.01$, * $p<0.05$, NS $P>0.05$, versus control, paired Student's t test. Data represent the mean ± SEM (**a**-**c**, **e**).

a

Lane	Expected size	PCR primers	Cas9/sgRNA transfection
1	DNA ladder	NEB 1kb Plus DNA ladder	NA
2	2423 bp	*SCN8A* del SVA F1 and R1	Cas9/sgRNA control
3	2423 bp	*SCN8A* del SVA F1 and R1	*CHAF1B* sgRNA set 1
4	515 bp	*SCN8A* del SVA F1 and R1	*SCN8A* sgRNA set 1
5	none	*SCN8A* del SVA F1 and R1	water

b 1 2 3 4 5

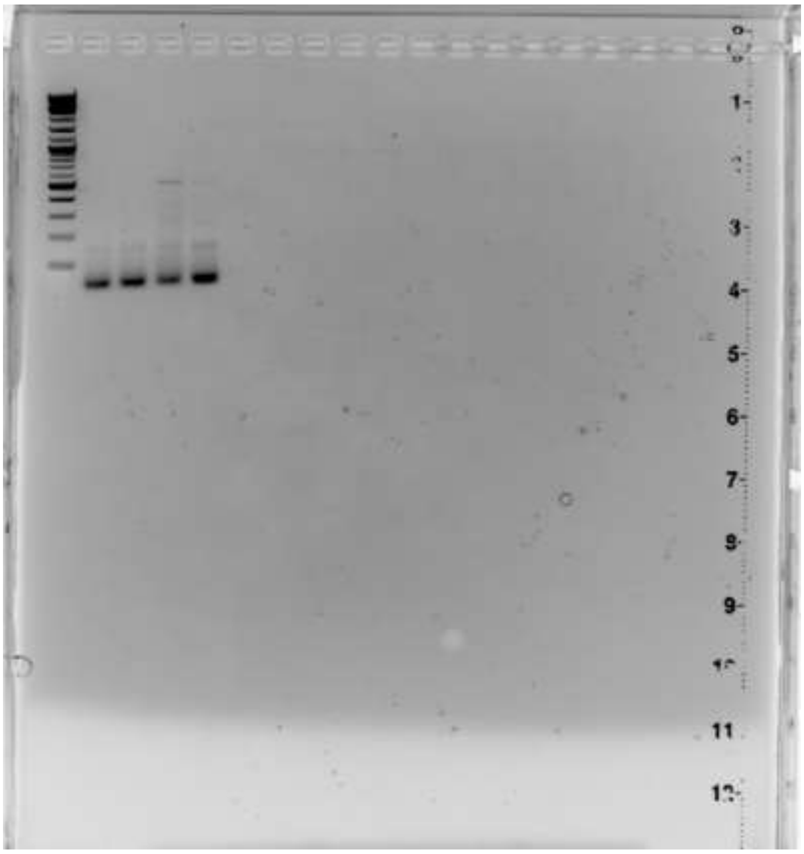

Supplementary Fig. 13: Complete gel image for deletion of the intronic SVA_D within *SCN8A*. Uncropped and unedited DNA agarose gel image used to generate **Fig. 4a** showing the expected PCR product size of 515 bp is produced with Cas9/sgRNAs used to delete the intronic SVA within the *SCN8A* gene. **a**, Table of the genomic samples and primers used in the PCRs to detect the deletion of the SVA within *SCN8A* shown in **b**. The sgRNA combination and sequences are in Supplementary Table 3 and the genomic PCR primer sequences are listed in Supplementary Table 4. *SCN8A* guides showed evidence of deletion of the SVA, lane 4. Note that neither the Cas9/sgRNA control nor the *CHAF1B* sgRNA set 1 (additional control) showed deletion. The undeleted PCR band (i.e., band spanning the SVA) was not visualized in lanes 2 and 3 because PCRs were optimized with a short PCR extension time.

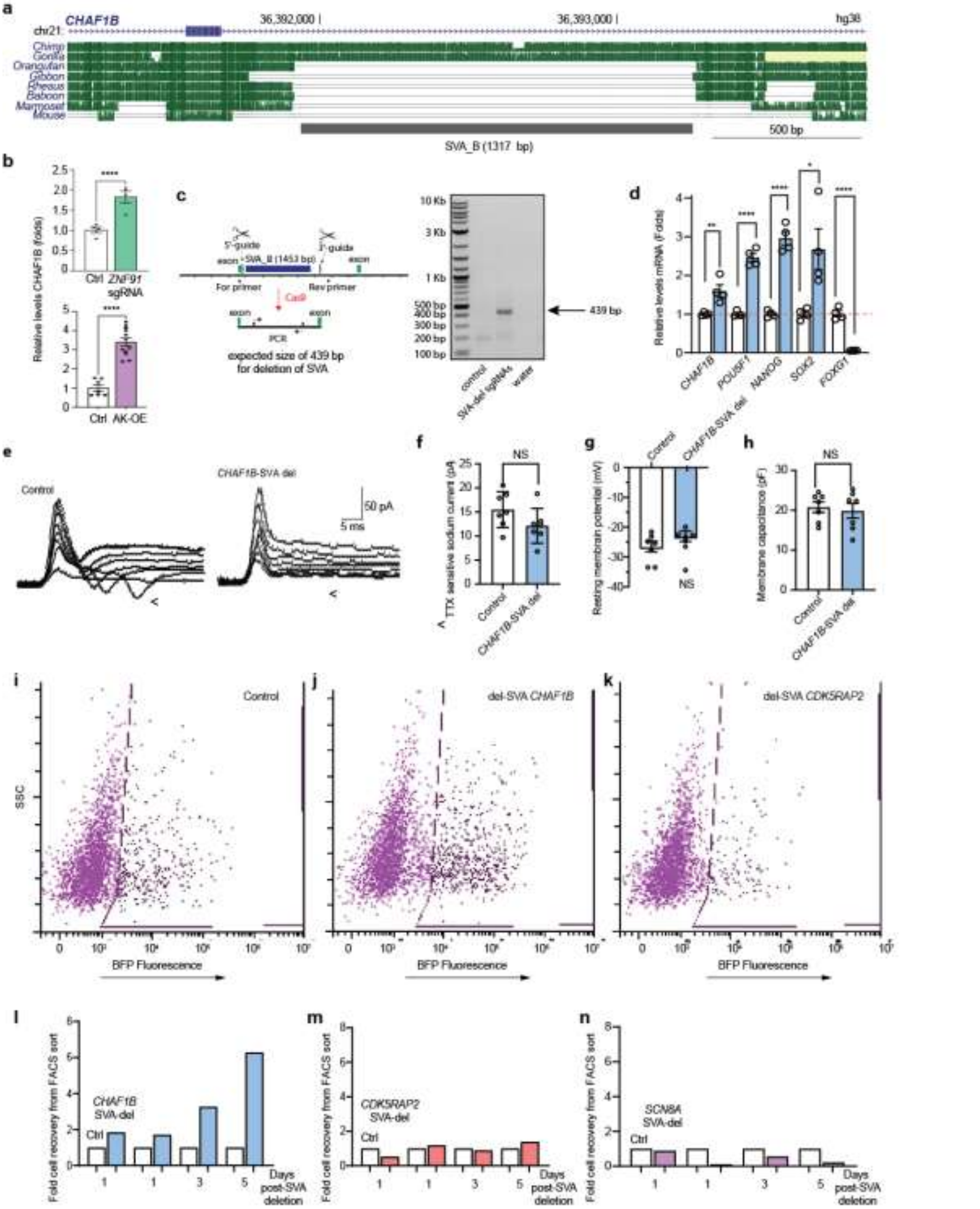

Supplementary Fig. 14: Deleting the SVA in *CHAF1B* increases stemness gene transcripts and overall cell number recovery. **a**, Schematic showing SVA_B located within the *CHAF1B* gene (UCSC genome browser, GRCh38/gh38). **b**, RT-qPCR of *CHAF1B* in NTera-2 cells transfected with SVA-lncRNA *AK057321* (AK-OE) vs. control plasmid or ZNF91 sgRNA/Cas9 vs. control, with *GAPDH* as the internal reference gene. Unpaired Student's two-tailed t test for individual gene comparisons to control. **c**, Strategy for Crispr/Cas9 deletion of the SVA_B within *CHAF1B* employed with DNA agarose gel image showing expected SVA-deletion PCR product size of 439 bp with sgRNAs cloned in tandem. **d**,

NTera-2 cells transfected with *CHAF1B* SVA-deletion sgRNAs were sorted and analyzed for gene expression by RT-qPCR with *GAPDH* as the internal reference gene. **e**, Representative sodium current traces in *CHAF1B*-SVA del group in response to step depolarization. **f**, TTX sensitive current in *CHAF1B*-SVA deleted (n=7) is not significantly different than controls (n=7). Unpaired Student's t test. **g**, Resting membrane potential (RMP) in *CHAF1B*-SVA deleted (n=7) and control (n=7) are not significantly different. Unpaired Student's t test. **h,** Capacitance in *CHAF1B*-SVA deleted (n=7) and control (n=7) are not significant different. Unpaired Student's t test. Data represent the mean ± SEM. **i-k**, Representative FACS sorting profiles for del-SVA *CHAF1B* (**j**), del-SVA *CDK5RAP2* (**k**) and control (**i**) sorted cells. The gating strategy for all sorting was based solely on fluorescence encoded in each transfected vector (x-axis), including control. **l-n**, Cell number recovery from each indicated sort from 4 independent experiments expressed as folds increase as compared to control = 1-fold. Note that days post transfection for each sort are indicated. Data represent the mean ± SEM.**** p<0.0001, *** p<0.001, ** p<0.01, * p<0.05, NS P>0.05, versus control.

a

**Lane**	**Expected size**	**PCR primers**	**Cas9/sgRNA transfection**
1	DNA ladder	NEB 1kb Plus DNA ladder	NA
2	2014 bp	*CHAF1B* del SVA F1 and R1	Cas9/sgRNA control
3	439 bp	*CHAF1B* del SVA F1 and R1	*CHAF1B* sgRNA set 1
4	none	*CHAF1B* del SVA F1 and R1	water
5	NA	NA	NA
6	NA	NA	NA
7	NA	NA	NA
8	DNA ladder	NEB 1kb Plus DNA ladder	NA

b

1 2 3 4 5 6 7 8

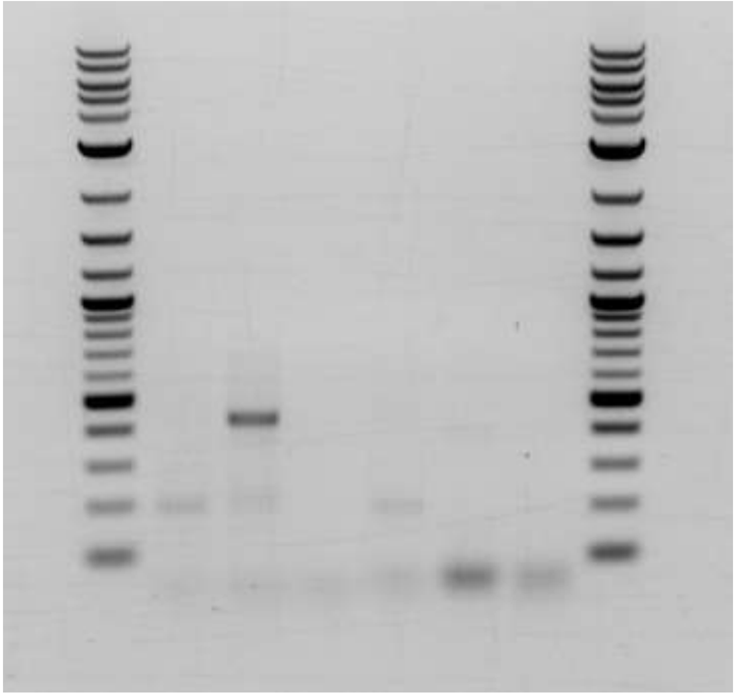

Supplementary Fig. 15: Complete gel image for deletion of the intronic SVA_B within

CHAF1B. Uncropped and unedited DNA agarose gel image used to generate Supplementary Fig. 15c, a DNA agarose gel image showing the expected PCR product size of 439 bp is produced with Cas9/sgRNAs designed to delete the intronic SVA within the *CHAF1B* gene. The sgRNA sequence combination is in the Supplementary Table 3 and the genomic PCR primer sequences are listed in Supplementary Table 4. *CHAF1B* guides showed evidence of deletion of the SVA, lane 4. The undeleted PCR band (i.e., band spanning the SVA) was not visualized in lane 2 because PCRs were optimized for a short PCR extension time. Lanes 5-7 are from PCRs designed to detect the deletion of a different intragenic SVA.

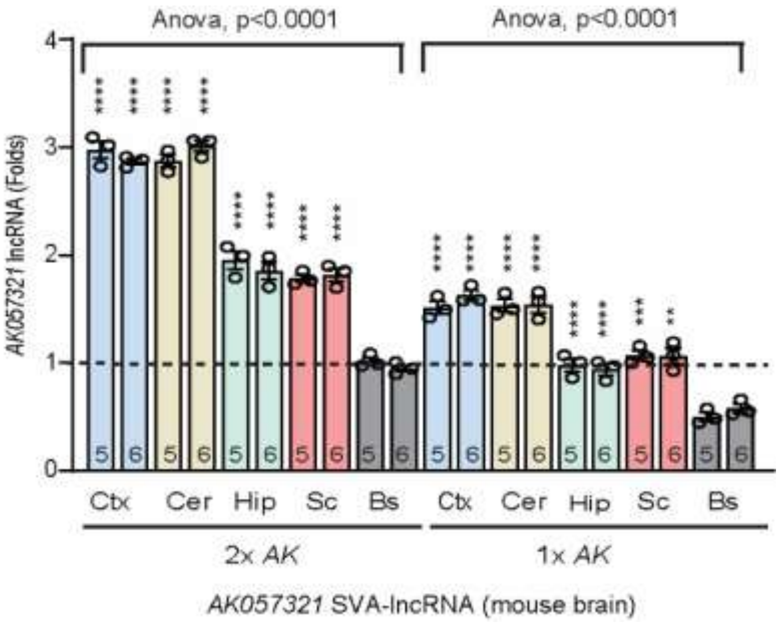

Supplementary Fig. 16: SVA-lncRNA *AK057321* expression is enriched in cortex and cerebellum relative to brainstem when expressed as a transgene in mouse in two independent transgenic mouse lines, 5 and 6. **a**, In 2x *AK057321* (homozygous) and 1x *AK057321* (heterozygous) mice, *AK057321* expression is increased more in cortex (Ctx), cerebellum (Cer), hippocampus (Hip) subcortex (Sc) compared to brainstem (Bs). One-way ANOVA ($F_{9,20}$=179.27, p<0.0001) in 2x *AK057321* (homozygous) mice with Bonferroni post hoc tests for each region compared to Bs. One-way ANOVA ($F_{9,20}$=51.29, p<0.0001) in 1x *AK057321* (heterozygous) with Bonferroni post hoc tests for each region compared to Bs. Note that in 2x *AK057321* (homozygous) compared to 1x *AK057321* (heterozygous) mice, *AK057321* expression is nearly doubled in expression within each region; one-way ANOVA ($F_{19,40}$=186.3, p<0.0001). Data represent the mean ± SEM. **** p<0.0001, *** p<0.001, ** p<0.01 versus Bs.

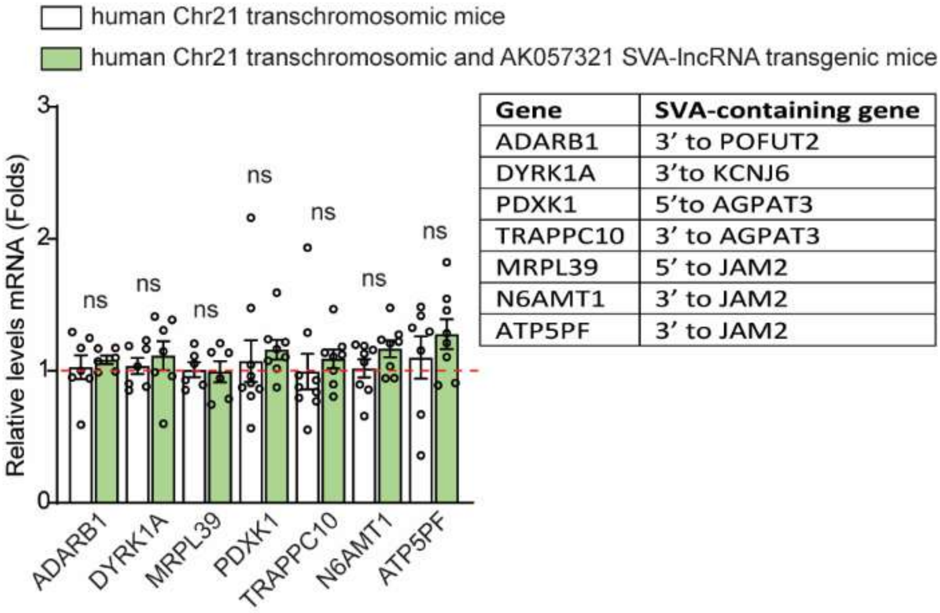

Gene	SVA-containing gene
ADARB1	3’ to POFUT2
DYRK1A	3’to KCNJ6
PDXK1	5’to AGPAT3
TRAPPC10	3’ to AGPAT3
MRPL39	5’ to JAM2
N6AMT1	3’ to JAM2
ATP5PF	3’ to JAM2

Supplementary Fig. 17: *AK057321* fails to regulated human chromosome 21 genes lacking SVAs. **a**, RT-qPCR expression levels of human genes nearby to the human genes with intragenic SVAs from mouse cortex of human Chr21 transchomosomic/1x *AK057321* transgenic mice compared to human Chr21 transchromosomic mice. B2M used as the internal reference gene. Unpaired Student’s t test. Data represent the mean ± SEM. ns $p>0.05$. **b**, Table indicates the SVA-containing gene closest to the neighboring gene analyzed.

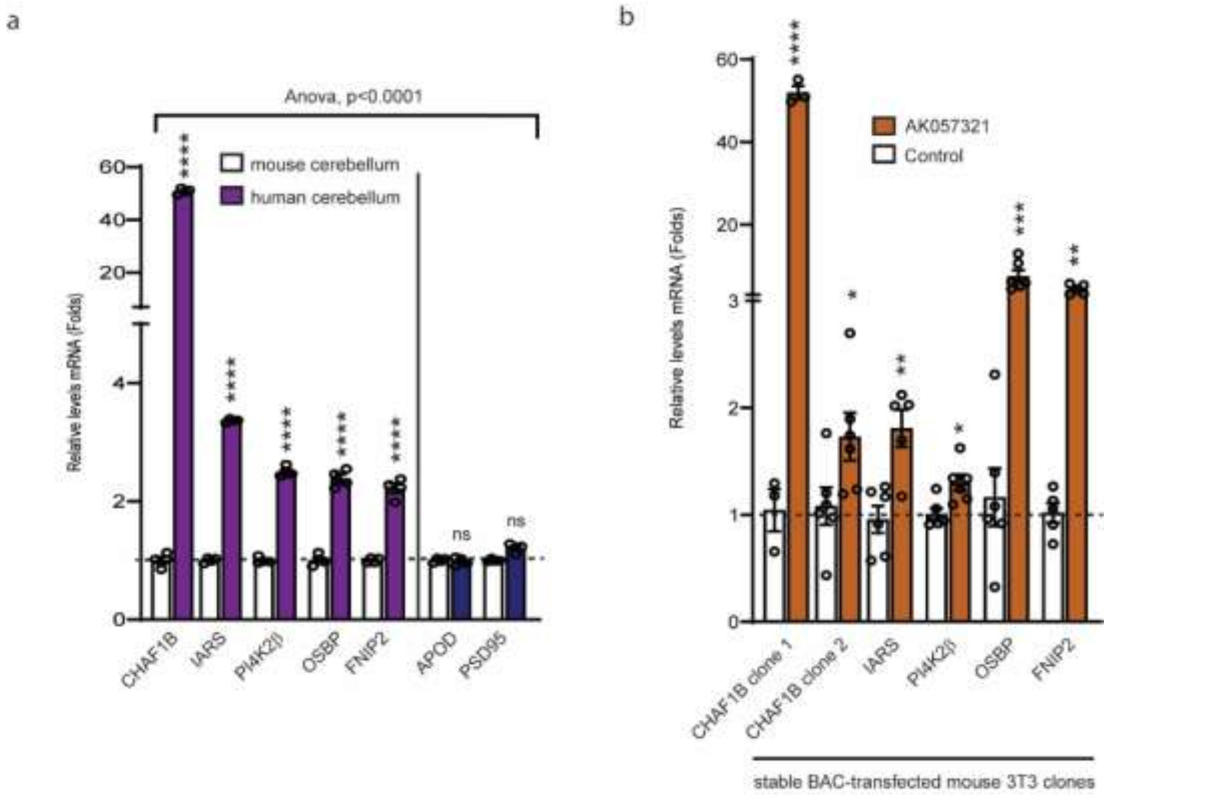

Supplementary Fig. 18: Multiple human genes with intronic SVAs are up-regulated in human relative to mouse cerebellum and are upregulated by SVA-lncRNA *AK057321* in mouse 3T3 fibroblast stably expressing these human genes (bacterial artificial chromosome, BAC vector). **a**, RNA was isolated from mouse or human cerebellum, and RT-qPCR was performed for each indicated gene. Two control genes that encode apolipoprotein D (APOD), a white matter restricted protein and postsynaptic density protein 95, a grey matter enriched synaptic protein, each display minimal differences between mouse and human cerebellum. Note that neither of the control genes has an SVA insert. One-way ANOVA ($F_{13,42}$=7.134, p<0.0001) with Bonferroni post hoc tests. **b**, Individual 3T3 clones stably transfected with the indicated BAC DNA were infected with either *AK057321* SVA-lncRNA or control virus followed by harvest, RNA extraction and RT-qPCR with Gapdh as the internal reference gene. Unpaired Student's t test. Data represent the mean ± SEM. **** p<0.0001, *** p<0.001, ** p<0.01, * p<0.05, ns p>0.05.

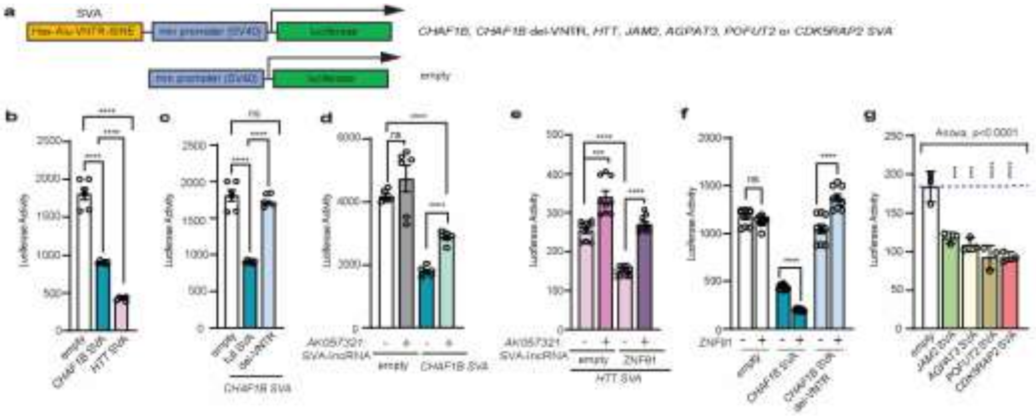

Supplementary Fig. 19: Transcriptional repression by intronic SVA transposons and ZNF91 transcription factor is reversed fully by VNTR repeat deletion (in *CHAF1B* SVA) and partially by SVA-lncRNA *AK057321*. **a**, Schematics representing structure of minimal luciferase reporter with or without added SVA transposon sequences. **b,** Luciferase activity using the *CHAF1B* SVA, *HTT* SVA reporter or empty reporter plasmid. **c**, Luciferase activity using the *CHAF1B* SVA, *CHAF1B del-VNTR* or empty reporter plasmid. **d**, Luciferase activity with the *CHAF1B* SVA or empty reporter plasmid and co-transfected with either *AK057321* SVA-lncRNA or control plasmid. **e**, Luciferase activity of the *HTT* SVA reporter plasmid co-transfected with *AK057321* SVA-lncRNA with and without *ZNF91*. **f,** Luciferase activity of the *CHAF1B* SVA, *CHAF1B SVA del-VNTR* or empty reporter plasmid co-transfected with and without *ZNF91*. **g**, Luciferase activity of other intronic SVAs, *JAM2*, *AGPAT3*, *POFUT2* and *CDK5RAP2* compared to the empty vector reporter. One-way ANOVA ($F_{4,10}$ =26.71, p<0.0001) versus empty vector reporter with Bonferroni post hoc tests. (**b-f**) Unpaired Student's t test. Data represent the mean ± SEM. **** p<0.0001, *** p<0.001, ** p<0.01, * p<0.05, ns p>0.05.

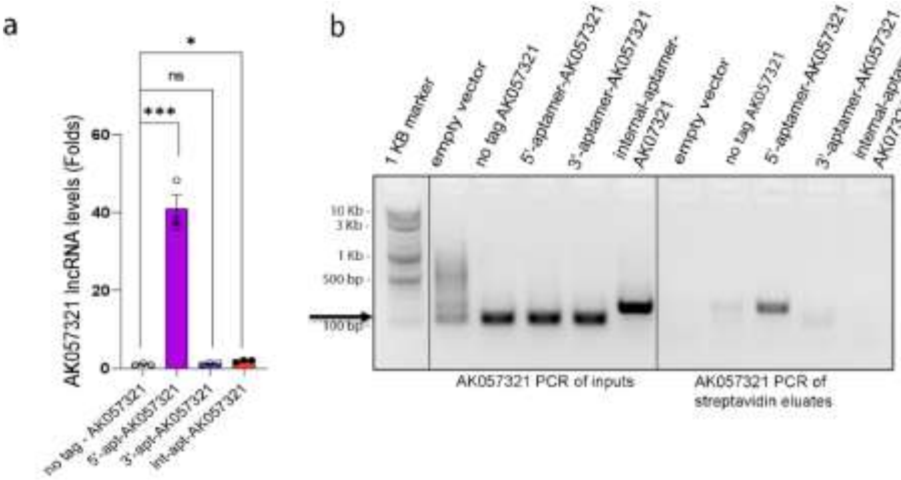

Supplementary Fig. 20: 5'-apatamer-tagged SVA-lncRNA *AK057321* binds to streptavidin beads. A dual-apatamer tag with affinity for streptavidin was inserted at three different locations in *AK057321*. HEK293T cells were transfected with each version and compared to un-tagged *AK057321*, followed by cell lysis, precipitation on streptavidin beads, elution by heating, and analysis for associated *AK057321* by RT-qPCR (**a**) and by RT followed by PCR and analysis of product(s) on a DNA agarose gel (**b**). Unpaired Student's t test. Data represent the mean ± SEM. *** p<0.001, * p<0.05, ns p>0.05.

a

Lane	Expected size	PCR primers	Input/pulldown
1	DNA ladder	NEB 1 kb Plus DNA ladder	NA
2	110 bp	*AK057321* FWD 1 & REV 1	empty vector
3	110 bp	*AK057321* FWD 1 & REV 1	*AK057321* – no tag
4	110 bp	*AK057321* FWD 1 & REV 1	*AK057321* - 5’ aptamer tag
5	110 bp	*AK057321* FWD 1 & REV 1	*AK057321* - 3’ aptamer tag
6	211 bp	*AK057321* FWD 1 & REV 1	*AK057321* - internal aptamer tag
7	110 bp	*AK057321* FWD 1 & REV 1	empty vector
8	110 bp	*AK057321* FWD 1 & REV 1	*AK057321* – no tag
9	110 bp	*AK057321* FWD 1 & REV 1	*AK057321* - 5’ aptamer tag
10	110 bp	*AK057321* FWD 1 & REV 1	*AK057321* - 3’ aptamer tag
11	211 bp	*AK057321* FWD 1 & REV 1	*AK057321* - internal aptamer tag
12	110 bp	*AK057321* FWD 1 & REV 1	empty vector
13	110 bp	*AK057321* FWD 1 & REV 1	*AK057321* – no tag
14	110 bp	*AK057321* FWD 1 & REV 1	*AK057321* - 5’ aptamer tag
15	110 bp	*AK057321* FWD 1 & REV 1	*AK057321* - 3’ aptamer tag
16	211 bp	*AK057321* FWD 1 & REV 1	*AK057321* - internal aptamer tag
17	DNA ladder	NEB 1 kb Plus DNA ladder	NA

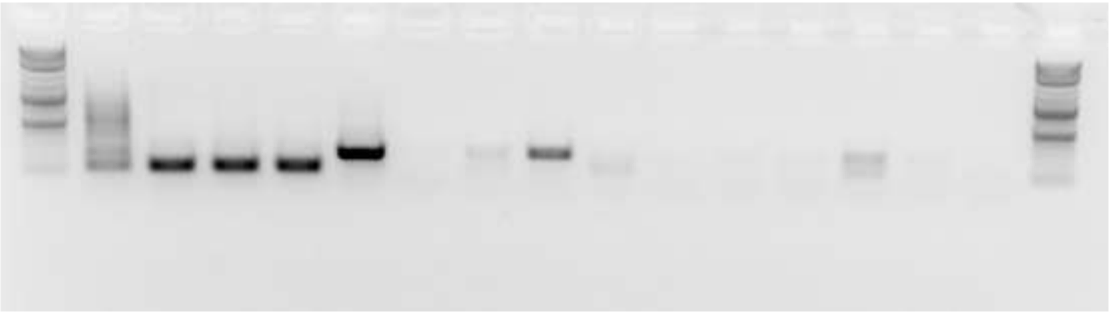

Supplementary Fig. 21: Complete gel image for 5'-apatamer-tagged SVA-lncRNA *AK057321* binds to streptavidin beads. **a**, Table indicating the primers used and samples analyzed in each lane of the uncropped and unedited DNA agarose gel image PCR bands shown in **b** that was used to generate Supplementary Fig. 21b. Primer sequences are listed in Supplementary Tables 5 and 16. PCR reactions are on lanes 1-6 are from the inputs prior to pulldowns, lanes 7-11 were pulldowns with streptavidin beads that were eluted with heat and lanes 12-16 are pulldowns with streptavidin beads that were eluted with biotin. Note that the internal aptamer tag results in a larger PCR band (lane 6) because the tag (extra 101 bp) is located within the PCR region spanned by the forward and reverse primers.

Supplementary Tables 1-20

Supplementary Table 1. SVA-lncRNA *AK057321* shRNA target sequences and cloning primers.

Oligo name	Oligo Sequence (5' – 3')
shRNA-1S	GACTATATCATCATGTACA
shRNA-1A	TGTACATGATGATATAGTC
shRNA-2S	CATCAAGATGCAGTCTCCC
shRNA-2A	GGGAGACTGCATCTTGATG
UbC Fwd	ttattatatcttgtggaaaggacgaggatccggaagaattcgagcggccgcgaATTAACCCGTGTCGGCTC
UbC Rev	tcgtggcgccagaGTCTAACAAAAAAGCCAAAAACG
P2A Fwd	ttggctttttgttagacTCTGGCGCCACGAATTTC
P2A Rev	ctggtcaacttggccatTCTAGACACCATAGGACCAG
Zeocin Fwd	tatggtgtctagaATGGCCAAGTTGACCAGTG
Zeocin Rev	cctcccctgaaccTCAGTCCTGCTCCTCGGC
T2A Fwd	gccgaggagcaggactgaGGTTCAGGGGAGGGAAGAG
T2A Rev	tcgcccttgctcaccatggtggcgaccggtTCTAGAGGTCATGGGTCC

Supplementary Table 2. Construction primers used to modify lentiCRISPRv2 hygro (Addgene #98291) to express in tandem sgRNAs with Cas9 and either mcherry or blue fluorescent protein.

Oligo name	Oligo Sequence (5' – 3')
mcherry for hygro Fwd	ccgaaaaagcctgaactcaccgcgacgtctgtcCCGGTCGCCACCATGGTG
mcherry for hygro Rev	atccagaggttgattgtcgacttaacgcgtCTAGTTACTTGTACAGCTCGTCCATGC
long guide Fwd	tccagtttggttaattaaggtaccTTCGGGTTTATTACAGGGACAGC
long guide Rev	taggccctcctcgagGATGTGCGCTCTGCCCAC
short guide Fwd	cagagcgcacatcctcgagGAGGGCCTATTTCCCATG
short guide Rev	aattcccactcctttcaagacctagcTCCTTTCAAGACCTAGCTAG
T2A Fwd	ggctgtgtagaagtacttcgccgaacgcgtGGTTCAGGGGAGGGAAGAG
T2A Rev	tccttaatcagctcgctTCTAGAGGTCATGGGTCC
BFP Fwd	catgacctctagaAGCGAGCTGATTAAGGAG
BFP Rev	atccagaggttgtcgacttaacgcgtTTAATTAAGCTTGTGCCC

Supplementary Table 3. sgRNA sequences used to delete SVAs.

Gene with SVA	Oligo name	Oligo Sequence (5' – 3')	In tandem cloning
AK057321	*AK057321* sgRNA 1 left	GACAGCAATTTAGCAGTATCTGG	Set 1
AK057321	*AK057321* sgRNA 1 right	GCTTTGGATACCTCGTAGGTGGG	Set 1
AK057321	*AK057321* sgRNA 1 left	GACAGCAATTTAGCAGTATCTGG	Set 2

AK057321	*AK057321* sgRNA 2 right	GGTCATCGTGCCCACCTACGAGG	Set 2
AK057321	*AK057321* sgRNA 1 left	GACAGCAATTTAGCAGTATCTGG	Set 3
AK057321	*AK057321* sgRNA 3 right	ATGAGCTTGAATAGTTATCTTGG	Set 3
CDK5RAP2	*CDK5RAP2* sgRNA 1 left	GGCTTAGATTATGGTGCATCAGG	Set 1
CDK5RAP2	*CDK5RAP2* sgRNA 1 right	GAGCTCCCAATGGCCAAGTCTGG	Set 1
CDK5RAP2	*CDK5RAP2* sgRNA 2 left	CCACGAGGGGTTTTATGCCCTGG	Set 2
CDK5RAP2	*CDK5RAP2* sgRNA 2 right	TGTTCAGATTGACCCAGACTTGG	Set 2
CDK5RAP2	*CDK5RAP2* sgRNA 3 left	GGATTTTATGCCTTGAGCCCTGG	Set 3
CDK5RAP2	*CDK5RAP2* sgRNA 3 right	CAATTTGAAGGAGCTCCCAATGG	Set 3
CDK5RAP2	*CDK5RAP2* sgRNA 4 left	TGAGGTTAGTGCTAGTCACTTGG	Set 4
CDK5RAP2	*CDK5RAP2* sgRNA 4 right	ACCTGCAACAGTTATTACTGTGG	Set 4
CDK5RAP2	*CDK5RAP2* sgRNA 5 left	TTCAAAGTATGACGAGAAGCAGG	Set 5
CDK5RAP2	*CDK5RAP2* sgRNA 5 right	GTTGTATTATGTCTACCATGAGG	Set 5
SCN8A	*SCN8A* sgRNA 1 left	GGGATCTTAGAAGGGATGCAGGG	Set 1
SCN8A	*SCN8A* sgRNA 1 right	AGTCTACAGGATGCCACTAATGG	Set 1
SCN8A	*SCN8A* gRNA 2 left	GCTGATCACAAGAAGTAGTGTGG	Set 2
SCN8A	*SCN8A* gRNA 2 right	TCAGGAACACATTAGTCTACAGG	Set 2
CHAF1B	*CHAF1B* gRNA left	ACTTCTAAATTAGACCAGCCTGG	Set 1
CHAF1B	*CHAF1B* gRNA right	TTTACGATGAGTGCATTAAATGG	Set 1
CHAF1B	*CHAF1B* gRNA left	ACTTCTAAATTAGACCAGCCTGG	Set 2
CHAF1B	*CHAF1B* gRNA right	GTAAACAGAGATCACTGCCATGG	Set 2

Supplementary Table 4. Genomic PCR primers used to screen for SVA deletions.

**Oligo name**	**Oligo Sequence (5' – 3')**
AK057321 del SVA F1	TCACAGGGTCTGACAATTCAAA
AK057321 del SVA R1	GTGTGTATCCTTATAGTTGAGCAG
CDK5RAP2 del SVA F 1	TGACCTTGACTGGTTTGAAGAG
CDK5RAP2 del SVA F 2	CAGGTGGTGAGAATATGGGAAA
CDK5RAP2 del SVA F 3	AGTCTTGGCTAATCTGGAACAG
CDK5RAP2 del SVA F 4	GTCGGAGTCAAAGGAATGAGAA
CDK5RAP2 del SVA F 5	GGCTTGTCTGGTGTTTCTCT
CDK5RAP2 del SVA R 1	GAGGGAGAACCTGCTTACTTATT
CDK5RAP2 del SVA R 2	GCCTGTCATCTCAGCACTTT
SCN8A del SVA F 1	GTTGTGTTAAGGGTCAACTGAATAG
SCN8A del SVA R 1	GTATTGTCTGTGGCTGCTTCT
CHAF1B del SVA F 1	AATCGCTTGAACCCAGGAG
CHAF1B del SVA R 1	CTGCAGATGCTGTCATCCTAT

Supplementary Table 5. A sephadex and a streptavidin dual aptamer tag.

**Dual Aptamer Tag Oligo Sequence (5' – 3')**
TCCGAGTAATTTACGTTTTGATACGGTTGCGGAACCGACCAGAATCATGCAA GTGCGTAAGATAGTCGCGGGCCGGG

Supplementary Table 6. Primers used to generate Tet-inducible *CDK5RAP2* cDNA.

Oligo name	Oligo Sequence (5’ – 3’)
TetO FWD	ATCTCGAGTTTACCACTCCCTATCAGTGATAGA
TetO REV	GAGCTCTGCTTATATAGGCCTCCCACCGTAC
CDK5RAP2 TetO HiFi For	gccttttgctggccttttgctcacatgtATCTCGAGTTTACCACTC
CDK5RAP2 TetO HiFi Rev	cgagatctgagtccggtagcgctGAGCTCTGCTTATATAGG

Supplementary Table 7. Primers used to clone *CHAF1B* SVA into pGL3 luciferase vector.

Primer name	Primer oligo sequence (5’-3’)
CHAF1B SVA FWD	CCTCTCCCTCTCCCTCTC
CHAF1B SVA REV	AGTATTTATTGATCATTCTTGGGTGTTTC
CHAF1B SVA FWD HiFi NEB assembly	atttctctatcgataggtaccCCTCTCCCTCTCCCTCTC
CHAF1B SVA REV HiFi NEB assembly	tcgcagatctcgagcccgggctagcAGTATTTATTGATCATTCTT GGGTGTTTC

Supplementary Table 8. Primers used to generate *CHAF1B* SVA minusVNTR sequence into pGL3 luciferase vector.

Primer name	Primer oligo sequence (5’-3’)
CHAF1B Part A SVA FWD	CCTCTCCCTCTCCCTCTC
CHAF1B Part A SVA REV	TTTGGGAGGCCAAGGCAG
CHAF1B Part B FWD	TGGGAGGTGTACCCAACAG
CHAF1B Part B REV	AGTATTTATTGATCATTCTTGGGTGTTTC
CHAF1B Part A SVA FWD HiFi NEB assembly	atttctctatcgataggtaccCCTCTCCCTCTCCCTCTC
CHAF1B Part A SVA REV HiFi NEB assembly	acacctcccaTTTGGGAGGCCAAGGCAG
CHAF1B Part B FWD HiFi NEB assembly	gcctcccaaaTGGGAGGTGTACCCAACAG
CHAF1B Part B REV HiFi NEB assembly	tcgcagatctcgagcccgggctagcAGTATTTATTGATCATT CTTGGGTGTTTC

Supplementary Table 9. Primers used to clone *HTT* SVA into pGL3 luciferase vector.

Primer name	Primer oligo sequence (5’-3’)
HTT SVA FWD	GCAGAATAATGCTCCCCTCT
HTT SVA REV	GGCAGCAGCATATACCGAGT
HTT SVA screen FWD	GCCAATTGTGCAGTTCCTTT
HTT SVA screen REV	GATACTCGCAGCCAACCATT

Supplementary Table 10. Primers used to clone *JAM2*, *AGPAT3*, *POFUT2* and *CDK5RAP2* SVAs into pGL3 luciferase vector.

Primer name	Primer oligo sequence (5’-3’)
JAM2 SVA FWD	CAGCCTTGTCAACACCTTTATTT
JAM2 SVA REV	TTTGTGGAGGAACACGATCTC
AGPAT3 SVA FWD	AACATCAGCACAGACCAAGAA
AGPAT3 SVA REV	GCTTTCTGCCTTTCAGACTTTG
POFUT2 SVA FWD	GGATCCTCCAGTAAGGACAGATA
POFUT2 SVA REV	CCACAGATGCCGTCAGAAA
CDK5RAP2 SVA FWD	CCGGGAGGATGGAGTTCA

CDK5RAP2 SVA REV	TTTGGAGGTGCGTAATGTCAG
JAM2 HiFi SVA FWD	ATTTCTCTATCGATAGGTACCCAGCCTTGTCAACACCTTTATTTC
JAM2 HiFi SVA REV	TCGCAGATCTCGAGCCCGGGCTAGCTTTGTGGAGGAACACGATC
AGPAT3 HiFi SVA FWD	ATTTCTCTATCGATAGGTACCAACATCAGCACAGACCAAG
AGPAT3 HiFi SVA REV	TCGCAGATCTCGAGCCCGGGCTAGCGCTTTCTGCCTTTCAGAC
POFUT2 HiFi SVA FWD	ATTTCTCTATCGATAGGTACCGGATCCTCCAGTAAGGAC
POFUT2 HiFi SVA REV	TCGCAGATCTCGAGCCCGGGCTAGCCCACAGATGCCGTCAGAAAG
CDK5RAP2 HiFi SVA FWD	ATTTCTCTATCGATAGGTACCCCGGGAGGATGGAGTTCAG
CDK5RAP2 HiFi SVA REV	TCGCAGATCTCGAGCCCGGGCTAGCTTTGGAGGTGCGTAATGTCAG
JAM2 HiFi SVA FWD	ATTTCTCTATCGATAGGTACCCAGCCTTGTCAACACCTTTATTTC

Supplementary Table 11. Table of BAC clone numbers used in this study.

**Gene**	**Clone ID**
CHAF1B	RP11-108J14
IARS	CTD-2055I8
PI4K2β	RP11-23L20
OSBP	CTD-2303H12
FNIP2	RP11-28O10

Supplementary Table 12. Table of primers used in BAC recombineering.

**Primer name**	**Oligo primer sequence (5’-3’)**
NeomycinR FWD	CTGAAGAGGAGTTTACGTCCAG
NeomycinR REV	ATTAAGGGTTCCGCAAGCTC
5’ homology arm to pBACe3.6 (RP11)	CGCATTAAAGCTTATCGATGATAAGCTGTCAAACATGAGAATTGATCCGG
Forward primer for neo insertion cassette (RP11)	CGCATTAAAGCTTATCGATGATAAGCTGTCAAACATGAGAATTGATCCGG*CTGAAGAGGAGTTTACGTCCAG*

3' homology arm to pBACe3.6 (RP11)	CTGCATCCGATGCAAGTGTGTCGCTGTCGACGGTGACCCTATAGTCGAGG
Reverse primer for neo insertion cassette (RP11)	<u>CTGCATCCGATGCAAGTGTGTCGCTGTCGACGGTGACCCTATAGTCGAGG</u>*ATTAAGGGTTCCGCAAGCTC*
5' homology arm to pBeloBAC11 (CTD)	TTCCTTTCTCTGTTTTTGTCCGTGGAATGAACAATGGAAGTCCGAGCTCA
Forward primer for neo insertion cassette (CTD)	<u>TTCCTTTCTCTGTTTTTGTCCGTGGAATGAACAATGGAAGTCCGAGCTCA</u>*CTGAAGAGGAGTTTACGTCCAG*
3' homology arm to pBeloBAC11 (CTD)	TACAATCTGCTCTGATGCCGCATAGTTAAGCCAGCCCCGACACCCGCCAA
Reverse primer for neo insertion cassette (CTD)	TACAATCTGCTCTGATGCCGCATAGTTAAGCCAGCCCCGACACCCGCCAAATTAAGGGTTCCGCAAGCTC

Supplementary Table 13. Table of BAC screening primers.

**Primer name**	**Primer oligo sequence (5'-3')**
RP11-neo-Seq-for	CGATGAGCGCATTGTTAGATTTC
RP11-neo-Seq-rev	GGTTATGTGGACAAAATACCTGGTT
CTD-neo-Seq-for	GACAGGTGCTGAAAGCGAG
CTD-neo-Seq-rev	CTGGCGTAATAGCGAAGAGG

Supplementary Table 14. Primers used in BAC recombineering to delete SVA in *CHAF1B* (BAC clone RP11-108J14).

Oligo name	Oligo Sequence (5' – 3')
CHAF1B left arm For	AGACAGTTAGCCAGGATGG
CHAF1B left arm Rev	TCCCTGTTGAAATGCTTGG
Zeocin For construction	TAGCTGGGACTACAGGTGCATCGGATCCACTAGTAACGGC
Zeocin Rev construction	TGTAATCCCAGCTACTCGGGCGCCAGTGTGATGGATATCTG
CHAF1B right arm For	TACAGGCCTGCACCACTATG
CHAF1B right arm Rev	CAGGCAGAGATTGCAGTGAG

Supplementary Table 15. Primers used to clone zeocin resistance gene.

Oligo name	Oligo Sequence (5' – 3')
Zeo Res For	TCGGATCCACTAGTAACGGC
Zeo Res Rev	CGCCAGTGTGATGGATATCTG

Supplementary Table 16. Table of IDT primers and probes used in gene expression studies.

Primer ID	Sequence (5'-3')
AK057321 FWD 1	GCTACTAGAACCACTGACTTCAT
AK057321 REV 1	GAGAATCAGGCAGGGATGTT
AK057321 PRB 1	5'/56-FAM/AAGATGCAG/ZEN/TCTCCCTCTGA
AK057321 FWD 2	GCTACTAGAACCACTGACTTCATC
AK057321 REV 2	AGGCTGAGGCAGGAGAA
AK057321 PRB2	5'/56-FAM/ACCATCTCG/ZEN/GCTCACTGCAACATC/3IABkFQ/3'
ZNF91 FWD[1]	CCAGACCTGATTAGTTATCTGG
ZNF91 REV[1]	ACATTTTTCATATTTTCTCAGTAATAC
CDK5RAP2	Prime Time Std qPCR Assay; Hs.PT.58.28138308
CDK5RAP2	Prime Time Std qPCR Assay; Hs.PT.58.22477909
NIPBL	Prime Time qPCR Primers; Hs.PT.58.4309431
SPATA5	Prime Time qPCR Primers; Hs.PT.58.5
ATRX	Prime Time Std qPCR Assay; Hs.PT.58.939584
WAPL	Prime Time Std qPCR Assay; Hs.PT.58.27929275
KDM6A	Prime Time qPCR Primers; Hs.PT.58.19290698
ANKDR11	Prime Time qPCR Primers; Hs.PT.27139934
CHAF1B FWD 1	GGGCACCGTTCTACTTCTTC
CHAF1B REV 1	TGACGGTGCCTCTGACT
CHAF1B PRB1	5'/56-FAM/TCCGGGTCC/ZEN/CTCCAGCATTT/3IABkFQ/3'
CHAF1B FWD 2	CATCTCCTCCCGSTGCTAAA
CHAF1B REV 2	TTTTTCCCCTTCGAGACTCA

CHAF1B PRB2	5’/56-FAM/TCTTGCTCG/ZEN/TCATACCAAAGCCGT/3IABkFQ/3’
RBL1	Prime Time qPCR Primers; Hs.PT.58.4123862
SCN8A	Prime Time Std qPCR Assay; Hs.PT.58.2478730
SCN8A	Prime Time Std qPCR Assay; Hs.PT.58.1641962
WNK3	Prime Time Std qPCR Assay; Hs.PT.58.25141664
KCNJ6	Prime Time qPCR Primers; Hs.PT.58.1954078
KCNH1	Prime Time Std qPCR Assay; Hs.PT.58.27443267
CASK	Prime Time Std qPCR Assay; Hs.PT.58.20249400
MYO5A	Prime Time Std qPCR Assay; Hs.PT.58.26606668
NRXN1	Prime Time Std qPCR Assay; Hs.PT.58.3713581
NRXN2	Prime Time Std qPCR Assay; Hs.PT.58.1397376
GRID1	Prime Time Std qPCR Assay; Hs.PT.58.19812755
SHANK2	Prime Time Std qPCR Assay; Hs.PT.58.2960615
ABL2	Prime Time qPCR Primers; Hs.PT.58a.3158121.g
CNTN4	Prime Time Std qPCR Assay; Hs.PT.58.20752389
NRCAM	Prime Time Std qPCR Assay; Hs.PT.58.2794163
MAPKAP1	Prime Time qPCR Primers; Hs.PT.58.24858668
RPTOR	Prime Time qPCR Primers; Hs.PT.58.26749458
BRAF1	Prime Time qPCR Primers; Hs.PT.56a.27823863
AGPAT3	Prime Time Std qPCR Assay; Hs.PT.58.28067831
LETM1	Prime Time Std qPCR Assay; Hs.PT.58.40993934
RANBP2	Prime Time Std qPCR Assay; Hs.PT.58.3984483
HUNK FWD	GGTGCCATCAGTTTCCTGCGC
HUNK REV	TGCCCGTGTAATTCTCATTAA
LSS FWD	CGCAAGGGTGGCTTCTCCTTCA
LSS REV	CTCAGCCGTGCAGTCAGAAAC
B2M	Prime Time Std qPCR Assay; Hs.PT.58v.18759587
GAPDH	Prime Time Std qPCR Assay; Hs.PT.39a.22214836
B2M	Prime Time qPCR Primers; Hs.PT.58v.18759587
GAPDH	Prime Time qPCR Primers; Hs.PT.39a.2214836
POU5F1	Prime Time Std qPCR Assay; Hs.PT.58.24436403
NANOG	Prime Time Std qPCR Assay; Hs.PT.58.21480849
SOX2	Prime Time Std qPCR Assay; Hs.PT.58.237787.g
POFUT2	Prime Time Std qPCR Assay; Hs.PT.58.24436403
PCBP3	Prime Time Std qPCR Assay; Hs.PT.58.25131904
CDK5RAP2	Prime Time Std qPCR Assay: Hs.PT.58.28138308
FOXG1	Prime Time Std qPCR Assay; Hs.PT.58.26906112.g
RWDD2B	Prime Time Std qPCR Assay; Hs.PT.58.4429456
JAM2	Prime Time Std qPCR Assay; Hs.PT.58.2739820
Pofut2	Prime Time Std qPCR Assay; Mm.PT.58.6904698
Chaf1b	Prime Time Std qPCR Assay; Mm.PT.58.12985685
Kcnj6	Prime Time Std qPCR Assay; Mm.PT.58.13521340
Agpat3	Prime Time Std qPCR Assay; Mm.PT.58.5384170
Pcbp3	Prime Time Std qPCR Assay; Mm.PT.14265841
Rwdd2b	Prime Time Std qPCR Assay; Mm.PT.12650934
Jam2	Prime Time Std qPCR Assay; Mm.PT.5384170
ADARB1 FWD	GCACAGATGTTAAAGATGCCA

ADARB1 REV	GATCTCCGAGATATTATTTCT
DYRK1A FWD	AGGTGCGTCAGCAATTTCCTG
DYRK1A REV	ACCTGTGTAGGAGCTTCAGTG
MRPL39 FWD	ATTTATAGCAACATCGCC
MRPL39 REV	CTTTCTCTTTATTAAAGAGAT
PDXK1 FWD	TGGCCTGTGAGAAGACCGTGT
PDXK1 REV	TGCATGGGGCTGGCCTCACT
TRAPCC10 FWD	TCGACAACAGTAGCAACTGGG
TRAPCC10 REV	GCAGCTTTTCCCACACACTGCCCA
N6AMT1 FWD	ACTGATATCAACCCTGAGGCA
N6AMT1 REV	ATAACTGGTTGAATGTGAACT
ATP5PF FWD	TGAAGATCCCAAATTTGAAGT
ATP5PF REV	GAACACACTCAACATCACCAA

Supplementary Table 17. Table of Eurofin primers used in gene expression studies. Primers that recognize both human and mouse transcripts for data shown in Supplementary Fig. 19 are bolded.

**Primer Name**	**Oligo Sequence (5'-3')**
RPL13A For	CGCTGTGAAGGCATCAACATTTC
RPL13A Rev	GCTGTCACTGCCTGGTACTTC
AK057321 For1	GGCTCCTTACCAGCTCACCT
AK057321 Rev1	CTCCTCACATCCCAGACGAT
AK057321 For2	ATGCAGTCTCCCTCTGATGC
AK057321 Rev2	CTCCTCACATCCCAGACGAT
HTT For	ACAAGCAAGAGACCCGAAGA
HTT Rev	GTCAGGGTTTGCAGAAGCTC
Htt For	GTCTAGTGCCCTTGCTCCAG
Htt Rev	ACCAACAGAACCAGGAGTGG
***CHAF1B* For**	GGGGCCACTTAGAAGATGTGTATGA
***CHAF1B* Rev**	ACTTCATGCTGTCGTCGTGAAAC
***IARS* For**	TGGATGCTTCAGGCTGCTTCAC
***IARS* Rev**	GGGGGTGCCCCAGTATCTGTTT
***PI4K2*β For**	TGACCGTGCAAAATCAAGAGGCAAA
***PI4K2*β Rev**	TTGAGGAAGCCAAGCCCAGTGAA
***OSBP* For**	ATCAACCTCGCCACAGCCAAC
***OSBP* Rev**	CTGGGCTAACATGAGGAAATCTCTGC
***FNIP2* For**	ACTGGAAGTAACCTAGCACACAGCA
***FNIP2* Rev**	CCTGTTCATGTGAGATTCAAACAGGGG
***APOD* For**	GAAGATGGTACGAAATTGAGAAGATC
***APOD* Rev**	TCGGTGGCCAGGATCCAGTA
***PSD95* For**	GTGACAACCAAGAAATACCGCTAC
***PSD95* Rev**	CCACCAGGAATGATCTTGGTGAT
***ACTB* For**	ACCCAGATCATGTTTGAGACCTTC
***ACTB* Rev**	CTTGCGCTCAGGAGGAGCAA
***GAPDH* For**	CATCACCATCTTCCAGGAGCG
***GAPDH* Rev**	CCACAGTCTTCTGGGTGGCA

Supplementary Table 18. Primers used for relative quantitative PCR (rqPCR) for *AK057321*

expression analysis in Supplementary Fig. 17.

**Oligo name**	**Oligo Sequence (5’ – 3’)**
AK057321 rqFor1	GGCTCCTTACCAGCTCACCT
AK057321 rqRev1	CTCCTCACATCCCAGACGAT
AK057321 rqFor2	ATGCAGTCTCCCTCTGATGC
AK057321 rqRev2	CTCCTCACATCCCAGACGAT

Supplementary Table 19. Thermocycler conditions for reverse transcriptase and polymerase chain reactions.

**Figures/Panels**	**Reaction Mix**	**Cycling Conditions**	**Instrument**
**1c**, **1g**, **1i-j**, **2b-c**, **3c-e**, **6a-e**, **7b**, **7i**, Supplementary Figs 2, 3, 13a-c, 15b, 15d, 18, 19b, 21a	M-MuLV Reverse Transcriptase (NEB M0254) with random primer mix (NEB S1330)	mix RNA with random primer mix and incubate at 65 °C for 5 minutes and chill on ice. Then add remaining components and incubate: 25°C 5 minutes, 42°C 60 minutes, 65°C 20 minutes; use immediately or store at -20°C	eppendorf Mastercycler nexus
**5b-e**, Supplementary Figs. 17, 19a	M-MLV Reverse Transcriptase (ThermoFisher 28025013)	mix RNA and primers and incubate at 65 °C for 5 minutes and chill on ice. Then add remaining components except enzyme and incubate at 37°C 2 minutes, and then add enzyme and incubate at 25 °C for 10 minutes, 37°C for 50 minutes, 70°C 15 minutes; use immediately or store at -20°C	eppendorf Mastercycler nexus
**1c**, **1g**, **1i-j**, **2b**, **3c-e**, **6a**, **6e**, **7b-e**, **7i**, Supplementary Figs. 2, 3, 13a-c, 13b, 13d, 21a	Taqman Gene Expression master mix (ThermoFisher; 4369016)	1. 50°C for 2 minutes<br>2. 95°C for 10 minutes<br>3. 95°C for 15 seconds<br>4. 60°C for 1 minute<br>5. GoTo 3 for 40 cycles	BioRad CFX384 Touch Real-Time PCR Detection System
**1g, 2c**, **5b-e**, **6b-d**,	PowerUp Sybr	1. 50°C for 2 minutes	BioRad CFX384

**7f-g**, Supplementary Figs. 17, 18, 19	Green Master Mix (ThermoFisher; A25779)	2. 95℃ for 2 minutes<br>3. 95℃ for 15 seconds<br>4. 60℃ for 1 minute<br>5. GoTo 3 for 40 cycles	Touch Real-Time PCR Detection System
**1h**, **3b**, **4a**, Supplementary Figs. 8, 12, 14, 15b, 16, 21b, 22	Azura 2X Taq Red Mix (Azura; AZ-1322)	1. 95℃ for 1 minute<br>3. 95℃ for 15 seconds<br>4. 55℃ for 15 seconds<br>5. 72℃ for 30 seconds<br>6. GoTo 3 for 35 cycles	eppendorf Mastercycler nexus
All cloning reactions excluding SVAs (requires high fidelity)	Phusion High Fidelity DNA Polymerase with HF buffer (NEB; M0530)	1. 98℃ for 30 seconds<br>3. 60℃ for 30 seconds<br>4. 72℃ for 15 seconds per kb<br>5. GoTo 3 for 35 cycles<br>6. 72℃ for 10 minutes	eppendorf Mastercycler nexus
SVA cloning reactions (performed on pure BAC clone templates containing each respective SVA; for luciferase assays shown in Supplementary Fig. 20	Phusion High Fidelity DNA Polymerase with GC buffer and 3% DMSO (NEB; M0530)	1. 98℃ for 30 seconds<br>3. 55℃ for 30 seconds<br>4. 72℃ for 30 seconds per kb<br>5. GoTo 3 for 35 cycles<br>6. 72℃ for 10 minutes	eppendorf Mastercycler nexus

Supplementary Table 20. Genomic IDT primers and probes used in SVA-lncRNA *AK057321* binding studies.

**Primer Name**	**Oligo Sequence (5’-3’)**
CHAF1B genomic SVA FWD	AAGATCATGCCACTGCACTC
CHAF1B genomic SVA REV	ACTGCGACCGGTCAGATATAG
CHAF1B genomic SVA PRB 1	/56-FAM/AGCTATAGC/ZEN/TAATATAGTGCCAGTGGA/3IABkFQ/
CDK5RAP2 genomic SVA FWD	GGAGTGTTATTGCCTCTAGGATT
CDK5RAP2 genomic SVA REV	CACACACACACACAGAGAATTG
SCN8A genomic SVA FWD	GTTGTGTTAAGGGTCAACTGAATAG
SCN8A genomic SVA REV	AGTTGGTTTCTCTGACAGGATT
