# Supplementary material for: Hominoid SVA-lncRNA AK057321 targets human-specific SVA retrotransposons in SCN8A and CDK5RAP2 to initiate neuronal maturation
Source: Commun Biol. 2023 Mar 30;6:347. doi: 10.1038/s42003-023-04683-8 (PMC10063665; doi:10.1038/s42003-023-04683-8)
Supplement: Supplementary file 3 — Description of Additional Supplementary Files [file 42003_2023_4683_MOESM3_ESM.pdf]

Description of Additional Supplementary Files

File name: Supplementary Data 1

Description: Duplication of SVA-lncRNA AK057321 in rare cases of autism spectrum disorder.

File name: Supplementary Data 2

Description: Human genes containing intronic SVAs

File name: Supplementary Data 3

Description: Gene ontology disease enrichment of human genes with intragenic SVAs.

File name: Supplementary Data 4

Description: Q Gene ontology term enrichment of human genes with intragenic SVAs.

File name: Supplementary Data 5

Description: RNAseq results of genes regulated with decreases of SVA-lncRNA AK057321 (shRNA) in NTera-2 cells.

File name: Supplementary Data 6

Description: Gene ontology term enrichment for biological processes of genes decreased 0.5-fold or more with decreases of SVA-lncRNA AK057321 (shRNA) in NTera-2 cells.

File name: Supplementary Data 7

Description: RNAseq results of genes regulated with SVA-lncRNA AK057321 overexpression in NTera-2 cells.

File name: Supplementary Data 8

Description: Gene ontology term enrichment for biological processes of genes up-regulated 1.5-fold or more by SVA-lncRNA AK057321 over-expression in NTera-2 cells.

File name: Supplementary Data 9

Description: Source data for main manuscript Figure graphs.

File name: Supplementary Data 10

Description: Source data for supplementary manuscript Figure graphs.
