# Supplementary material for: Hominoid SVA-lncRNA AK057321 targets human-specific SVA retrotransposons in SCN8A and CDK5RAP2 to initiate neuronal maturation
Source: Commun Biol. 2023 Mar 30;6:347. doi: 10.1038/s42003-023-04683-8 (PMC10063665; doi:10.1038/s42003-023-04683-8)
Supplement: Supplementary file 10 — Reporting Summary [file 42003_2023_4683_MOESM10_ESM.pdf]

nature portfolio

Corresponding author(s): Matthew P. Anderson

Last updated by author(s): Feb 20, 2023

Reporting Summary

Nature Portfolio wishes to improve the reproducibility of the work that we publish. This form provides structure for consistency and transparency in reporting. For further information on Nature Portfolio policies, see our Editorial Policies and the Editorial Policy Checklist.

Statistics

For all statistical analyses, confirm that the following items are present in the figure legend, table legend, main text, or Methods section.

n/a	Confirmed	
☐	☒	The exact sample size (*n*) for each experimental group/condition, given as a discrete number and unit of measurement
☐	☒	A statement on whether measurements were taken from distinct samples or whether the same sample was measured repeatedly
☐	☒	The statistical test(s) used AND whether they are one- or two-sided<br>*Only common tests should be described solely by name; describe more complex techniques in the Methods section.*
☒	☐	A description of all covariates tested
☒	☐	A description of any assumptions or corrections, such as tests of normality and adjustment for multiple comparisons
☐	☒	A full description of the statistical parameters including central tendency (e.g. means) or other basic estimates (e.g. regression coefficient) AND variation (e.g. standard deviation) or associated estimates of uncertainty (e.g. confidence intervals)
☐	☒	For null hypothesis testing, the test statistic (e.g. *F*, *t*, *r*) with confidence intervals, effect sizes, degrees of freedom and *P* value noted<br>*Give P values as exact values whenever suitable.*
☒	☐	For Bayesian analysis, information on the choice of priors and Markov chain Monte Carlo settings
☒	☐	For hierarchical and complex designs, identification of the appropriate level for tests and full reporting of outcomes
☒	☐	Estimates of effect sizes (e.g. Cohen's *d*, Pearson's *r*), indicating how they were calculated

Our web collection on statistics for biologists contains articles on many of the points above.

Software and code

Policy information about availability of computer code

Data collection	Supplemental data showing RNAseq: RNA library preparations, sequencing reactions and analysis were conducted at GENEWIZ, LLC. (South Plainfield, NJ, USA).
Data analysis	GO term enrichment analysis was performed at the following websites: pantherdb.org and g:Profiler – a web server for functional enrichment analysis and conversions of gene lists (ut.ee)

For manuscripts utilizing custom algorithms or software that are central to the research but not yet described in published literature, software must be made available to editors and reviewers. We strongly encourage code deposition in a community repository (e.g. GitHub). See the Nature Portfolio guidelines for submitting code & software for further information.

Data

Policy information about availability of data

All manuscripts must include a data availability statement. This statement should provide the following information, where applicable:

- Accession codes, unique identifiers, or web links for publicly available datasets
- A description of any restrictions on data availability
- For clinical datasets or third party data, please ensure that the statement adheres to our policy

All data generated or analyzed during this study are included in this published article (and its supplementary information files). Source data is available for all figures.

Human research participants

Policy information about studies involving human research participants and Sex and Gender in Research.

Reporting on sex and gender	Postmortem brain samples are from males
Population characteristics	Postmortem brain samples are from individuals aged 50-65
Recruitment	Postmortem brain samples were collected randomly from sequential cases with next-of-kin consent
Ethics oversight	Beth Israel Deaconess Medical Center IRB approve the human brain tissue studies

Note that full information on the approval of the study protocol must also be provided in the manuscript.

Field-specific reporting

Please select the one below that is the best fit for your research. If you are not sure, read the appropriate sections before making your selection.

☒ Life sciences ☐ Behavioural & social sciences ☐ Ecological, evolutionary & environmental sciences

For a reference copy of the document with all sections, see nature.com/documents/nr-reporting-summary-flat.pdf

Life sciences study design

All studies must disclose on these points even when the disclosure is negative.

Sample size	Group sizes were chosen based on adequate statistical power to detect differences.
Data exclusions	No data were excluded.
Replication	Experiments were independently replicated at least twice. For mice, a biological n indicates tissue from an independent animal with the same genotype. For cells, a biological n indicates cells from an independent set of transfection/FACS sort with the same vectors.
Randomization	All cell samples and animals were randomized.
Blinding	Blinding was performed during data collection and analysis.

Reporting for specific materials, systems and methods

We require information from authors about some types of materials, experimental systems and methods used in many studies. Here, indicate whether each material, system or method listed is relevant to your study. If you are not sure if a list item applies to your research, read the appropriate section before selecting a response.

Materials & experimental systems

n/a	Involved in the study	
☐	☒	Antibodies
☐	☒	Eukaryotic cell lines
☒	☐	Palaeontology and archaeology
☐	☒	Animals and other organisms
☒	☐	Clinical data
☒	☐	Dual use research of concern

Methods

n/a	Involved in the study	
☒	☐	ChIP-seq
☐	☒	Flow cytometry
☒	☐	MRI-based neuroimaging

Antibodies

Antibodies used	Anti-beta 3 Tubulin (TuJ1) Antibody (2G10, Santa Cruz) was used for staining TuJ1 and Alexa-conjugated secondary antibodies (Invitrogen) were used. The S9.6 RNA:DNA heteroduplex antibody was purchased from ThermoFisher.
Validation	All antibody validation information is available on the Santa Cruz, Invitrogen and FisherScientific websites along with associated literature.

Eukaryotic cell lines

Policy information about cell lines and Sex and Gender in Research

Cell line source(s)	NTera-2, NIH3T3 and HEK293T cells were all purchased from ATCC.
Authentication	Validated cell lines were purchased from ATCC and were not authenticated by us.
Mycoplasma contamination	Cell lines were purchased mycoplasma free and were not tested for mycoplasma by us.
Commonly misidentified lines (See ICLAC register)	None of the cell lines used are known to be misidentified on the ICLAC register.

Animals and other research organisms

Policy information about studies involving animals; ARRIVE guidelines recommended for reporting animal research, and Sex and Gender in Research

Laboratory animals	Human mutant HTT transgenic mice bearing an HD model mutation and carried in a yeast artificial chromosome vector, YAC128 mice, were purchased from the Jackson Laboratory, strain number 004938. AK057321 and CHAF1B transgenic mice were produced at the BIDMC transgenic core facility, as described in the methods.
Wild animals	This study did not involve wild animals.
Reporting on sex	Our findings do not apply to only one sex. Both female and male mice were used for gene expression studies.
Field-collected samples	This study did not involve samples collected from the field.
Ethics oversight	All protocols were approved by the BIDMC IACUC and Harvard Medical Area Standing Committee on Animals and the BIDMC IRB.

Note that full information on the approval of the study protocol must also be provided in the manuscript.

Flow Cytometry

Plots

Confirm that:

☒ The axis labels state the marker and fluorochrome used (e.g. CD4-FITC).

☒ The axis scales are clearly visible. Include numbers along axes only for bottom left plot of group (a 'group' is an analysis of identical markers).

☒ All plots are contour plots with outliers or pseudocolor plots.

☒ A numerical value for number of cells or percentage (with statistics) is provided.

Methodology

Sample preparation	NTera-2 cells were plated at a density of 5000 cells/cm3 12-16 h prior to transfection with the indicated plasmids using Lipofectamine 3000 (ThermoFisher) for the indicated times. Cells were harvested and FACS sorting of NTera-2 transfected fluorescent cells was performed at the BIDMC Flow cytometry Core.
Instrument	MOFLO Astrios EQ and on a BD FACSAria II
Software	BD FACs DIVA software
Cell population abundance	Post-sort fractions only contained cells that were distinctly shifted above that of the negative gating control (cells that were plated and harvested at the same time but were not transfected with the fluorescent plasmid).
Gating strategy	We did not gate based on FSC/SSC, but rather on fluorescence from transfected vectors that were expressing in each cell sample, compared to that of a control without a fluorescent vector in each experiment.

☒ Tick this box to confirm that a figure exemplifying the gating strategy is provided in the Supplementary Information.
